# PGRN protects against serum deprivation-induced cell death by promoting the ROS scavenger system in cervical cancer

**DOI:** 10.1038/s41419-024-07233-0

**Published:** 2024-12-18

**Authors:** Tingting Feng, Xiaoying Xu, Xiao Wang, Wei Tang, Yi Lu

**Affiliations:** 1 Department of Pathogenic Biology, School of Basic Medical Sciences, Shandong University, Jinan, Shandong China; 2 Biomedical Sciences College & Shandong Medicinal Biotechnology Centre, Shandong First Medical University & Shandong Academy of Medical Sciences, Jinan, Shandong China; 3 Shandong Provincial Maternal and Child Health Care Hospital Affiliated to Qingdao University, Jinan, Shandong China; 4 Department of Pathology, School of Basic Medical Sciences, Shandong University, Jinan, Shandong China; 5 Department of Biochemistry and Molecular Biology, School of Basic Medical Sciences, Shandong University, Jinan, Shandong China

**Keywords:** Cancer microenvironment, Mechanisms of disease

## Abstract

Progranulin (PGRN), an autocrine growth factor with tumorigenic roles in a variety of tumors, is a putative survival factor for normal and cancer cells in vitro. However, the fundamental mechanism of PGRN-mediated survival of cancer cells suffering from various types of microenvironmental stresses, such as serum deprivation, remains unknown. We show here that serum deprivation decreases intracellular PGRN protein levels in cervical cancer cells. PGRN protects cervical cancer cells against serum deprivation-induced apoptosis, limits reactive oxygen species (ROS) levels, maintains mitochondria integrity, and reduces oxidative damage of protein, lipid and DNA. PGRN enhances the ROS scavenger system, as evidenced by increased superoxide dismutase (SOD), catalase protein expression and activity, elevated GSH and NADPH levels and increased phase II detoxification enzyme expression in cervical cancer cells after serum withdrawal. The role of PGRN in ROS clearance is mediated by the PGRN-stimulated nuclear factor erythroid-derived 2-like 2 (NFE2L2)-antioxidant response element (ARE) pathway. Our study reveals an antioxidant role of PGRN in supporting the survival of cervical cancer cells under oxidative stress. This insight provides a new perspective on the how cervical cancer cells adapt to microenvironmental stress, contributing to cell viability and other malignant characteristics.

## Introduction

Cervical cancer is the most common cancer in women in 25 countries. In 2022, a total of 348,186 women died from cervical cancer [1]. Despite advancements in treatments such as radiotherapy and chemotherapy, the recurrence and distal metastasis rates of cervical cancer remain high [2]. Therefore, there is an urgent need to identify new therapeutic targets and develop effective treatment strategies to improve the prognosis of patients with cervical cancer.

Resisting cell death is one of the hallmarks of cancer cells [3]. However, cancer cells often suffer from stressful microenvironments, such as low oxygen pressure (i.e., hypoxia), and partial or complete lack of nutrients (e.g., hypoglycemia) and growth factors (e.g., low serum), which result from insufficient support from blood [4, 5]. Nutrient deprivation, especially glucose deficiency, is a universal phenomenon in solid tumors attributed to poor and/or a competing blood supply, especially in the center of a tumor during metastasis when cells detach from the vasculature to migrate [6]. This also triggers oxidative stress and contributes to cancer lesions by directly diminishing ATP production and increasing reactive oxygen species (ROS) production [7]. Growth factor deprivation or oncogenic kinase inhibition, also causes metabolic stress and increase oxidative stress [8]. Although specific ROS levels are required for normal cell signaling, high ROS levels can lead to cellular damage, p53 activation, induction of proapoptotic proteins, and cell death in a wide variety of tissues [9]. Compared with normal cells, cancer cells possess an enhanced antioxidant capacity to adapt to excessive ROS [10]. However, the mechanism by which cancer cells achieve antioxidant capacity is not fully understood [9, 11, 12]. Progranulin (PGRN), also known as acrogranin, proepithelin, or GP88/PC cell-derived growth factor, is an autocrine growth factor with multiple functions that has been implicated in various physiologic and disease processes [13, 14]. PGRN was originally identified as a growth factor for cancer cells and is strongly believed to mediate tumorigenesis in tumors including breast, lung, osteosarcoma, and liver cancer [15–17]. Guerra et al. reported that hypoxia and reduced extracellular pH promote PGRN expression in fibroblasts and that PGRN is cytoprotective to acidotic stress [18]. Our previous study revealed that PGRN protects against renal ischemia/reperfusion injury in mice and inhibits renal cell apoptosis induced by hypoxia and reoxygenation [19]. In cervical cancer models, we demonstrated that PGRN is overexpressed in cervical cancer cells and tissues and contributes to cervical cancer tumorigenesis both in vitro and in vivo [20]. Like other cancers, cervical cancer cells are subjected to various stresses during cancer progression. Adaptation to and tolerance of these microenvironments are key point in the cervical cancer progression. Therefore, our present study aimed to elucidate the role of PGRN in cervical cancer, particularly in extreme microenvironments. Our results revealed that PGRN protected cervical cancer cells from oxidative stress. We identified for the first time that PGRN promotes the nuclear translocation and transcriptional activity of nuclear factor (erythroid-derived 2)-like 2 (NFE2L2) during serum deprivation, through which PGRN enhances the ROS scavenging system. A better understanding of PGRN’s function in the context of microenvironmental stress may uncover new therapeutic opportunities for cervical cancer treatment.

## Results

### Serum deprivation reduces intracellular PGRN protein levels in cervical cancer cells

**Fig. 1:**
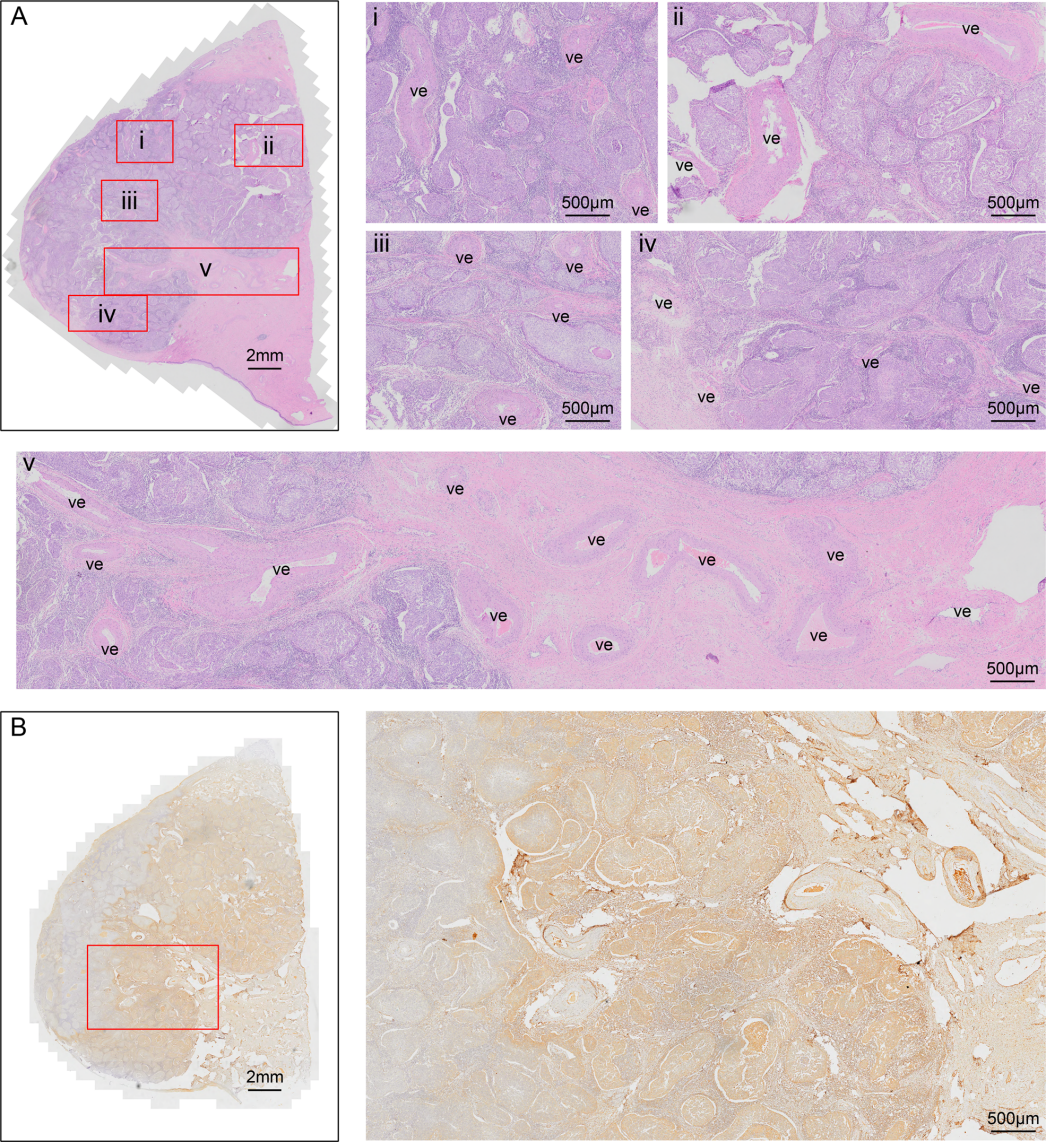
Increased progranulin (PGRN) protein levels in adjacent blood vessels in cervical cancer tissues. **A**, **B** The correlation between PGRN expression and vascular distribution in cervical cancer tissues was detected by hematoxylin and eosin staining (**A**) and immunohistochemistry (IHC) staining (**B**). ve vessels.

**Fig. 2:**
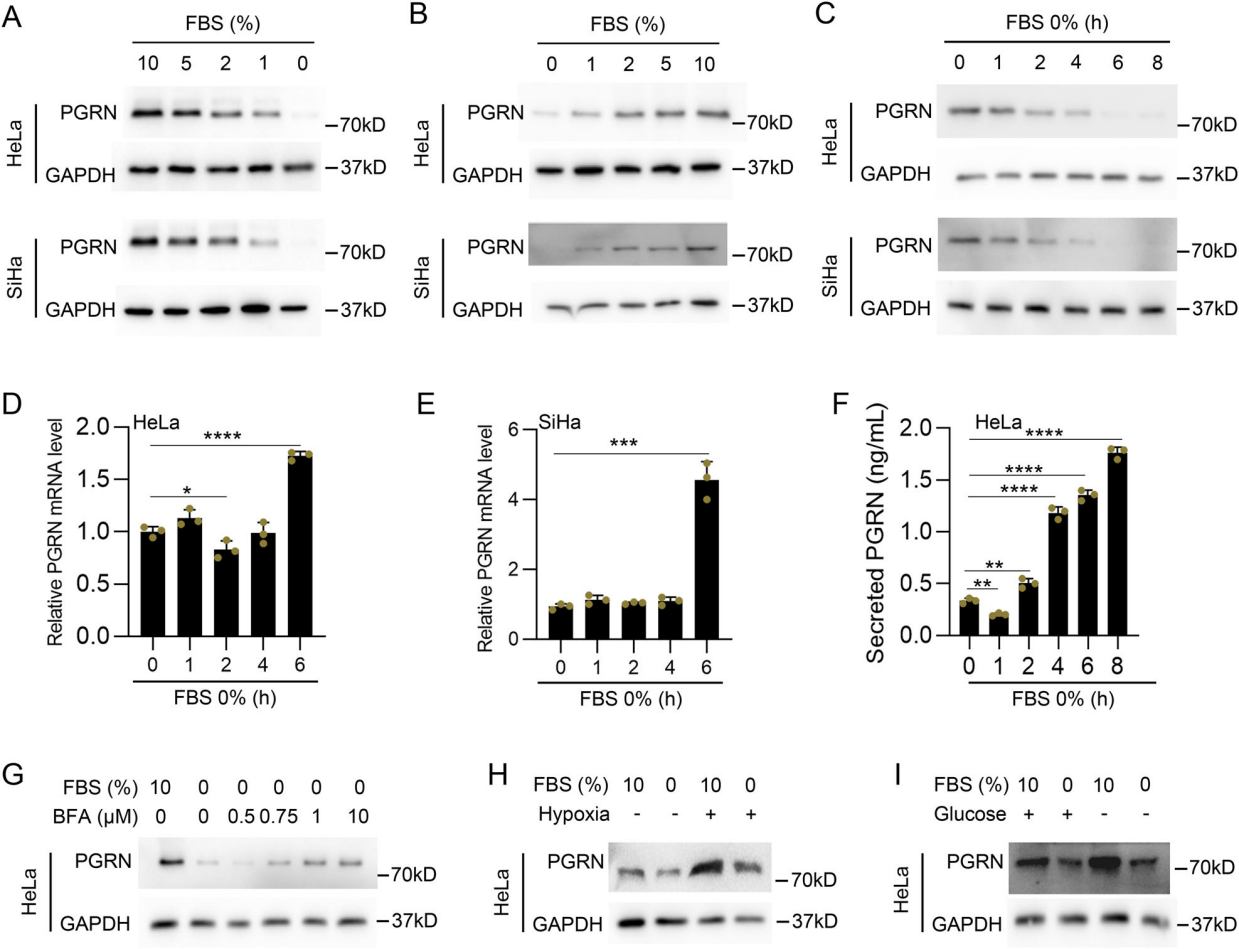
Serum deprivation reduces the intracellular PGRN protein levels. **A** HeLa and SiHa cells were pretreated with medium containing 10% FBS for 12 h and then treated with indicated serum concentrations for 6 h and intracellular PGRN protein levels was analyzed by western blotting. **B** HeLa and SiHa cells were pretreated with serum deprivation medium for 12 h and then treated with indicated serum concentrations for 6 h and intracellular PGRN protein levels was analyzed by western blotting. **C** HeLa and SiHa cells were treated with serum deprivation medium for indicated time and intracellular PGRN protein levels was analyzed by western blotting. **D**, **E** HeLa (**D**) and SiHa (**E**) cells were treated with serum deprivation medium for the indicated time. RNAs were collected and analyzed by qRT-PCR. Each experiment was performed with 3 replicates. **F** HeLa cells were treated with serum deprivation medium for indicated time. Secreted PGRN levels were analyzed by ELISA assay. **G** HeLa cells were treated with indicated concentration of BFA in the presence of serum deprivation for 6 h. Intracellular PGRN protein were analyzed by western blotting. **H** HeLa cells were treated with containing 0% or 10% FBS DMEM medium in the presence or absence hypoxia conditions, and intracellular PGRN protein levels was analyzed by western blotting. **I** HeLa cells were treated with containing 0% or 10% FBS high glucose DMEM / low glucose DMEM medium and intracellular PGRN protein levels was analyzed by western blotting. Data are presented as means ± SDs and are representative of three independent experiments. *P < 0.05, **P < 0.01, ***P < 0.001, ****P < 0.0001.

We first detected PGRN protein levels in cervical cancer tissues. As shown in Fig. 1A, B, there was a gradual decrease in PGRN levels in tumor nests distant from blood vessels, indicating that insufficient support from blood decreases PGRN protein levels in cervical cancer tissues. Next, we detected PGRN levels in the HeLa and SiHa cell lines under serum deprivation conditions. Western blot (WB) assays revealed that serum deprivation rapidly decreased intracellular PGRN protein levels in HeLa and SiHa cells. The reduction of fetal bovine serum (FBS) from 10% to 0% led to a dose-dependent decreased in intracellular PGRN protein levels in cervical cancer cells pretreated with 10% FBS (Fig. 2A), and addition of FBS from 0% to 10% enhanced PGRN protein levels in 0% FBS-pretreated cells (Fig. 2B). In addition, the PGRN protein level also decreased in a time-dependent manner in 0% FBS-treated HeLa, SiHa, MCF-7, HepG2 and A549 cells (Fig. 2C and Sup. Figure 1A). In contrast to the decrease in intracellular PGRN protein levels, PGRN mRNA levels were significantly increased in HeLa and SiHa cells at 6 h after serum deprivation (Fig. 2D, E), suggesting that the rapid decline of intracellular PGRN protein levels induced by serum deprivation is not due to changes in PGRN mRNA. Our data also showed that autophagy inhibitors 3-methyladenine (3-MA), Bafilomycin A1 (Baf-A1) and chloroquine (CQ), lysosomal inhibitor NH_4_Cl and lysosomal protease inhibitor leupeptin, as well as proteasome inhibitor MG132, were unable to prevent the decrease of intracellular PGRN protein levels in serum-deprived HeLa cells, demonstrating that the protein stability of PGRN is not involved (Supplementary Fig. 1B–D). Interestingly, the ELISA results of extracellular PGRN levels in conditioned medium indicated that the levels of PGRN secretion increased in a time-dependent manner in HeLa cells treated with 0% FBS (Fig. 2F). This result indicates that the decline of intracellular PGRN protein levels induced by serum deprivation is attributed to rapid PGRN secretion, which is also supported by the evidence that the inhibition of protein secretion by Brefeldin A (BFA) partially rescued the serum deprivation-induced decrease of intracellular PGRN protein in HeLa cells (Fig. 2G). In addition to serum deprivation, we also investigated the effects of hypoxia and hypoglycemia resulting from insufficient blood support on intracellular PGRN protein levels. As shown in Fig. 2H, I, hypoxia or glucose deprivation enhanced PGRN protein levels in HeLa cells, which were decreased when combined with serum deprivation, suggesting that decrease of PGRN level in tumor nests distanced from blood vessels is mainly caused by serum deprivation.

### PGRN protects against serum deprivation-induced cancer cell death

**Fig. 3:**
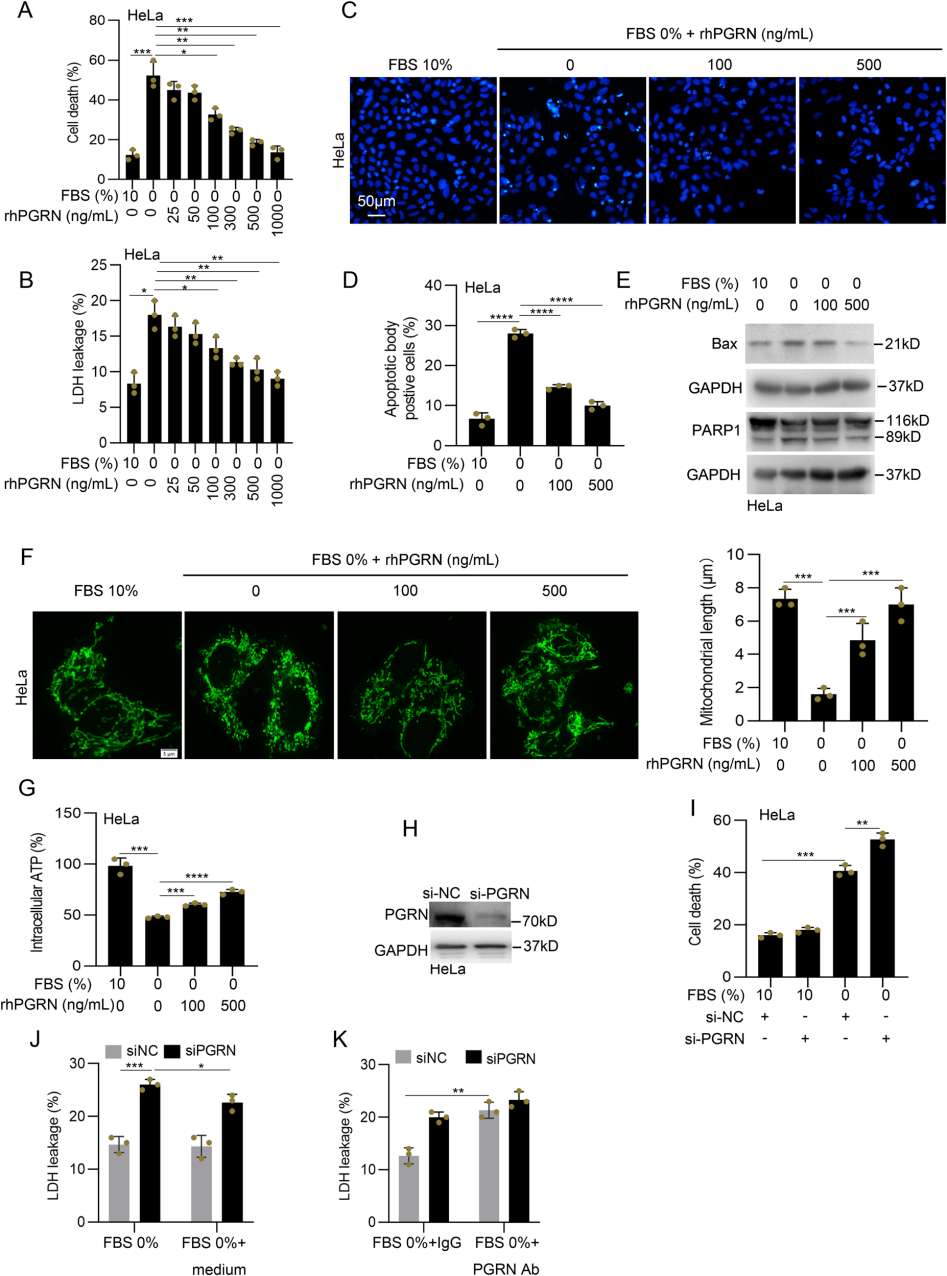
PGRN protects against serum deprivation-induced cancer cell death. **A** HeLa cells were treated with serum deprivation for 12 h and then treated with different concentration rhPGRN. Cell death was determined by trypan blue staining. **B** HeLa cells were treated as described in (**A**), and the percentage LDH leakage was determined by LDH assay. **C**, **D** Cells were treated as described in (**A**), and cell apoptosis was confirmed by DAPI staining. **E** Cells were treated as described in (**A**), and the levels of the apoptosis-associated proteins Bax and cleaved PARP1 were determined via western blotting. **F** Cells were treated as described in (**A**), mitochondrial staining with MitoTracker Green. **G** Cells treated as in (**A**), intracellular ATP production was measured. **H** The efficient knockdown of PGRN was determined by western blotting. **I** HeLa cells were transfected with PGRN siRNA, cell death was determined by trypan blue staining. **J**, **K** HeLa cells were transfected with siPGRN for 48 h and then treated with conditioned medium from 0% FBS-cultured cells (**J**) or PGRN antibody (**K**), the percentage LDH leakage was determined by LDH assay. Ab antibody. Data were presented as means ± SDs and are representative of 3 independent experiments. *P < 0.05, **P < 0.01, ***P < 0.001, ****P < 0.0001.

We next determined the role of PGRN in serum deprivation-induced cell death. Trypan blue staining assays indicated that recombinant human PGRN (rhPGRN) protected against the death of serum-deprived HeLa cells (Fig. 3A). In addition, the protective effect of PGRN on cells under serum-deprived conditions was confirmed using breast cancer MCF-7 and lung cancer A549 cell lines (Sup. Figure 2A-2B). rhPGRN administration rescued the lactate dehydrogenase (LDH) leakage from HeLa cells with serum deprivation (Fig. 3B), indicating the benefit of PGRN in serum deprivation-induced cytotoxicity. rhPGRN treatment reduced HeLa cell apoptosis in response to serum deprivation as evidenced by a decrease apoptotic body positive cells measured by DAPI staining (Fig. 3C, D) rhPGRN treatment also reduced the levels of proapoptotic Bax and cleaved PARP1 (Fig. 3E). Cells were labeled with the mitochondrial-specific dye Mitotracker Green, and a high degree of mitochondrial fragmentation was observed in serum-deprived HeLa cells, and this was effectively reversed upon rhPGRN treatment (Fig. 3F). Moreover, rhPGRN treatment inhibited H_2_O_2_ production induced by serum deprivation in HeLa cells (Sup. Figure 2C). In addition, rhPGRN dose-dependently increased intracellular ATP levels in serum-deprived HeLa cells (Fig. 3G). Conversely, HeLa cells transfected with a specific PGRN siRNA exhibited significantly reduced levels of PGRN and resulted in an increased death of HeLa cells induced by serum deprivation (Fig. 3H, I). In addition, the inhibition of PGRN expression led to enhanced serum deprivation-induced LDH leakage in HeLa cells, which was partially alleviated by the addition of conditioned medium from 0% FBS-treated HeLa cells (Fig. 3J). The addition of an antibody against PGRN exacerbated LDH leakage in HeLa cells under serum deprivation conditions (Fig. 3K). These data suggest that secreted PGRN protects against serum deprivation-induced cervical cancer cell death.

### PGRN ameliorates the ROS levels in serum-deprived cancer cells

**Fig. 4:**
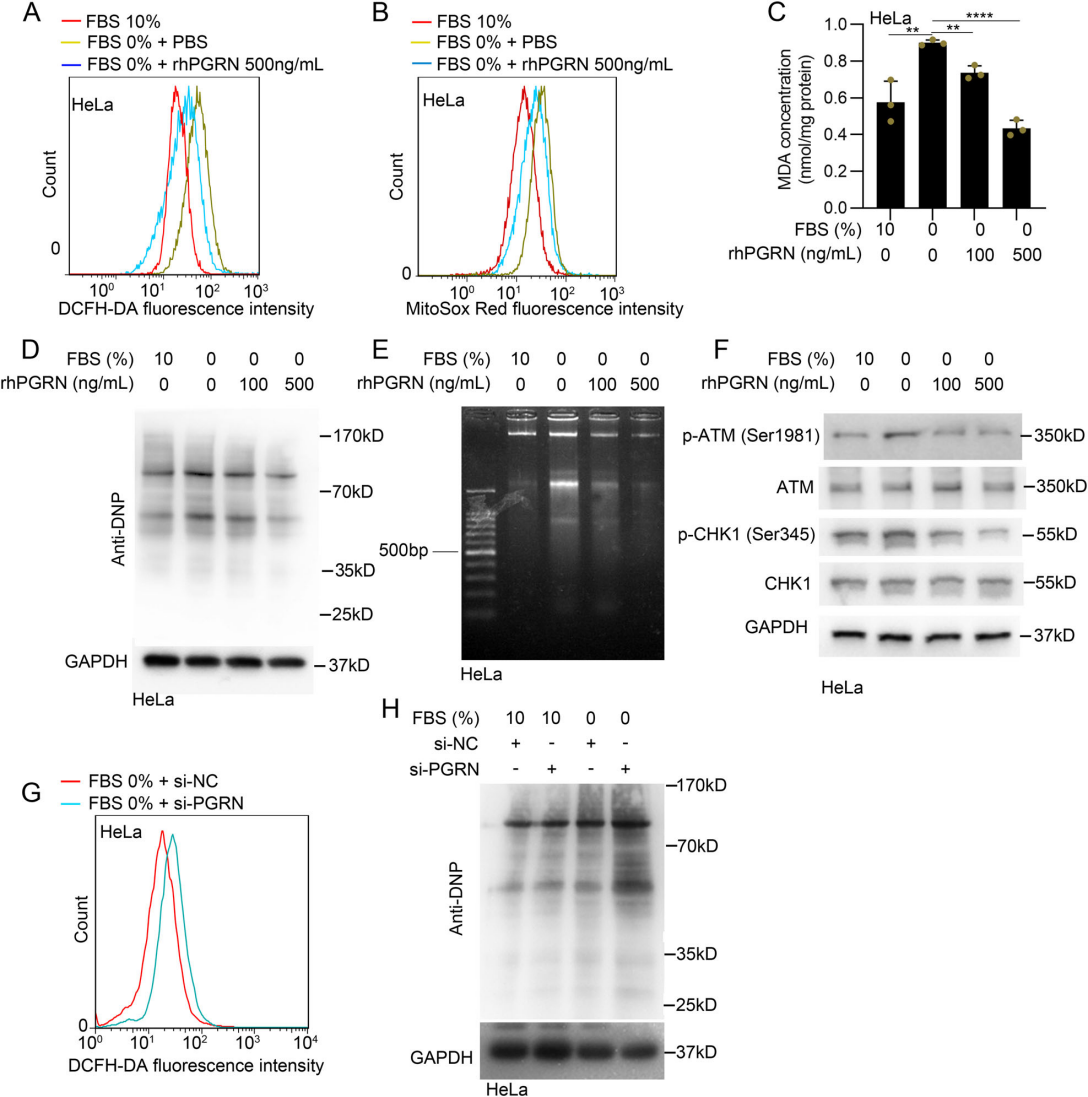
PGRN ameliorates the ROS levels of serum-deprived cancer cells. **A**, **B** HeLa cells were cultured with 10% FBS medium or treated with serum deprivation for 12 h and then treated with indicated concentration of rhPGRN. ROS and mitochondrial superoxide level were determined by DCFH-DA (**A**) and MitoSox Red (**B**) respectively. **C** Cells treated as in (**A**). Lipid peroxidation level were determined by using lipid peroxidation assay. **D** Cells treated as in (A). The protein oxidation level was determined by DNP antibody. **E** Cells treated as in (**A**), and DNA was extracted and analyzed on a 1.5% agarose gel. **F** Cells were treated as described in (**A**), and total ATM, total CHK1, p-ATM and p-CHK1 protein levels was analyzed by western blotting. **G** HeLa cells were transfected with PGRN siRNA. ROS levels were determined by DCFH-DA. **H** HeLa cells were transfected with PGRN siRNA for 48 h and then cultured with 0% or 10% FBS medium. The protein oxidation levels were determined by DNP antibody. Data are presented as means ± SDs and are representative of three independent experiments. **P < 0.01, ****P < 0.0001.

The production of ROS can be induced by serum deprivation and excessive ROS production damages cellular components such as DNA, proteins and lipids to induce cell death [9]. Given that PGRN promotes the survival of cervical cancer cells under serum deprivation conditions, we further investigated the role of PGRN in scavenging ROS. 2’,7’-dichlorofluorescein diacetate (DCFH-DA) staining revealed that rhPGRN inhibited the elevation of cellular ROS in serum-deprived HeLa cells (Fig. 4A). MitoSOX Red staining also revealed that rhPGRN disrupted the serum deprivation-induced increase in mitochondrial ROS levels (Fig. 4B). The production of malondialdehyde (MDA), a lipid peroxidation end product, was elevated in serum-deprived HeLa cells, which was markedly reduced by rhPGRN in a dose-dependent manner (Fig. 4C). Carbonylated proteins, a marker of severe protein oxidation, were detected with anti-dinitrophenyl (DNP) antibody after derivatization of carbonyl group with 2,4-dinitrophenylhydrazine (DNPH) [21]. As shown in Fig. 4D, rhPGRN dose-dependently inhibited serum deprivation-induced oxidation of protein. Excessive ROS also induce DNA damage, as we found decreased DNA laddering (Fig. 4E) in rhPGRN-treated HeLa cells during serum deprivation in comparison with PBS-treated cells. In response to DNA damage, DNA damage repair pathways, such as ATM-CHK2 and ATR-CHK1 axis, are activated. As shown in Fig. 4F, rhPGRN inhibits the levels of phosphorylated ATM and CHK1. Conversely, knocking down the expression of PGRN led to more cellular ROS accumulation (Fig. 4G) and protein oxidation (Fig. 4H) in serum-deprived HeLa cells.

### PGRN promoted the activities of the ROS scavenger system in serum-deprived cervical cancer cells

**Fig. 5:**
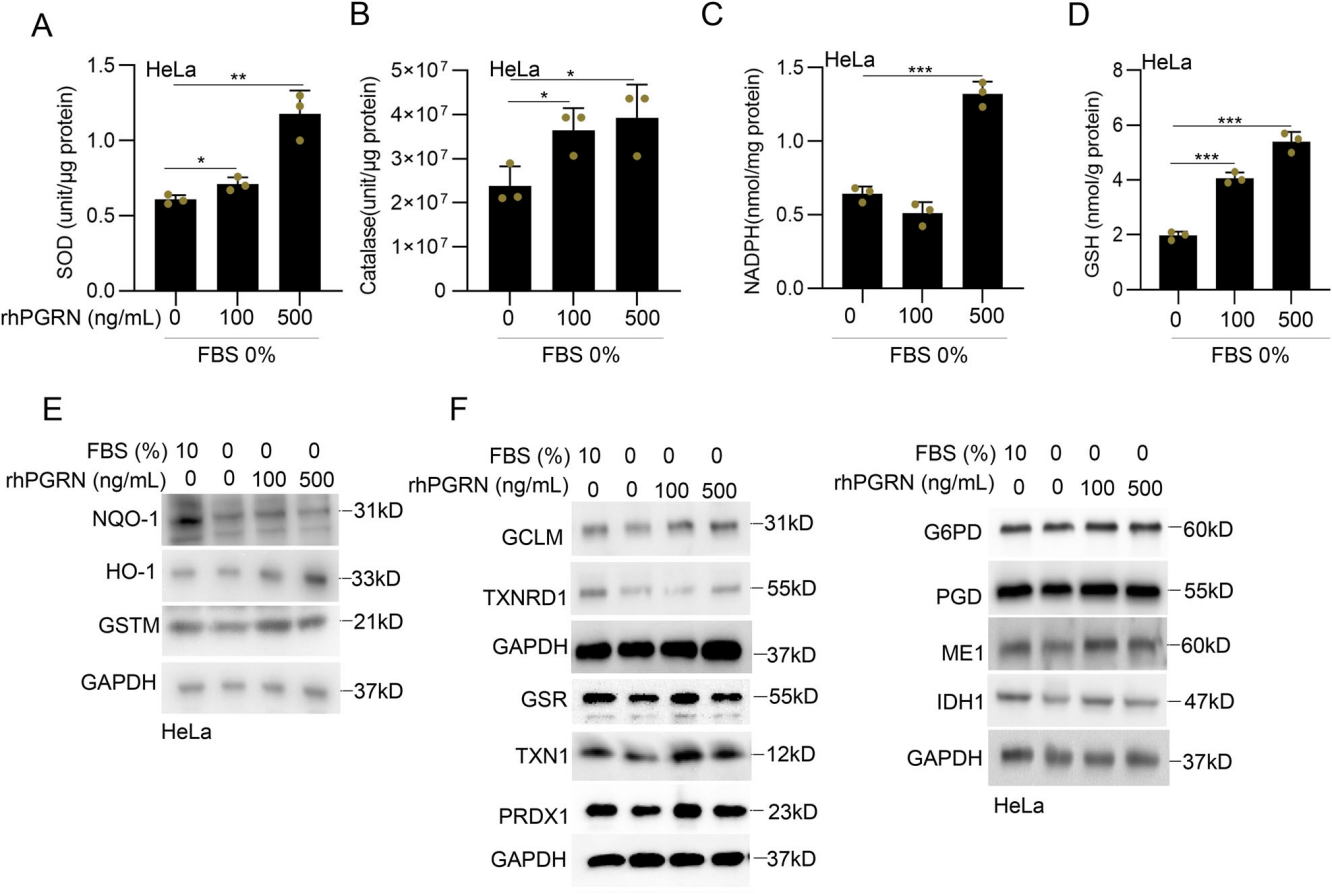
PGRN promoted the activities of the ROS scavenger system in serum-deprived cervical cancer cells. **A**–**D** HeLa cells were treated with serum deprivation for 12 h and then treated with indicated concentration of rhPGRN. The activity of SOD (**A**), catalase (**B**), NADPH (**C**) and GSH (**D**) were measured. **E**, **F** Cells were treated as described in (**A**), and phase II detoxification related protein (NQO-1, HO-1 and GSTM), GSH-related protein (GCLM and GSR), NADPH-related protein (G6PD, PGD, ME1 and IDH1) and TXN related protein (TXN1, TXNRD1, and PRDX1) were analyzed by western blotting. Data were presented as means ± SDs and are representative of three independent experiments. *P < 0.05, **P < 0.01, ***P < 0.001.

To explore the mechanism of the antioxidant role of PGRN in cervical cancer cells subjected to serum deprivation, the ROS scavenging system was analyzed. The activities of total superoxide dismutase (SOD) and catalase were enhanced by 500 ng/mL rhPGRN treatment in serum-deprived HeLa cells (Fig. 5A, B). rhPGRN increased the levels of intracellular reduced nicotinamide adenine dinucleotide phosphate (NADPH) (Fig. 5C) and reduced glutathione (GSH) levels (Fig. 5D) in serum-deprived HeLa cells. The protein levels of numerous phase II detoxifying enzymes (Fig. 5E) and antioxidant enzyme (Fig. 5F) of ROS scavenger system in serum-deprived HeLa cells were enhanced by rhPGRN treatment. Similarly, knocking down PGRN expression in HeLa cells resulted in reduced glucose-6-phosphate dehydrogenase (G6PD), glutamate-cysteine ligase modifier subunit (GCLM) and thioredoxin-1 (TXN1) protein levels (Fig. S3A). Inhibition of PGRN expression further decreased the intracellular GSH and NADPH levels in HeLa cells after serum deprivation (Fig. S3B, C).

### PGRN promoted the transcriptional activity of NFE2L2 in serum-deprived cervical cancer cells

**Fig. 6:**
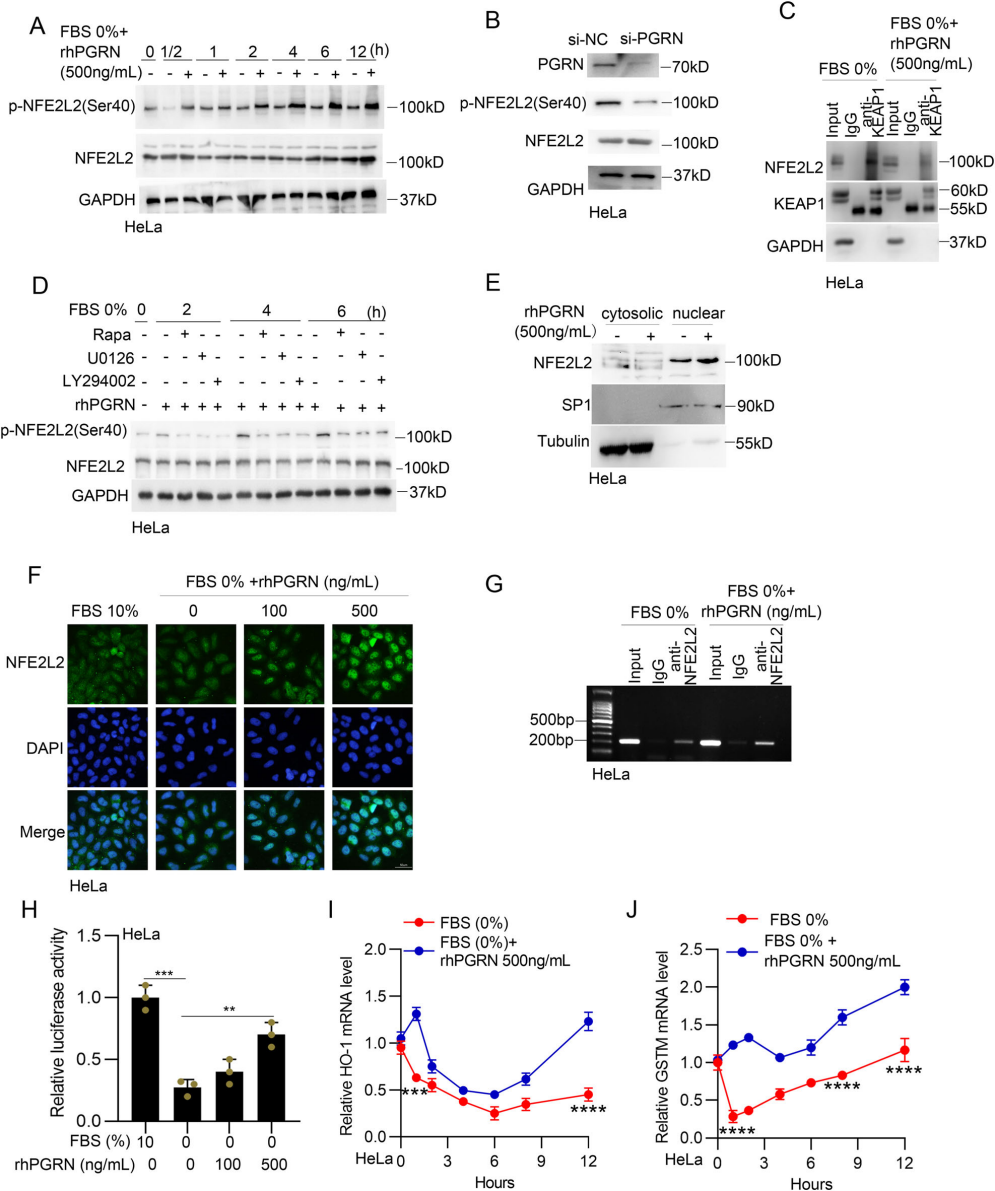
PGRN promoted NFE2L2/ARE pathway activation in serum-deprived cervical cancer cells. **A** HeLa cells were treated with serum deprivation medium in the absence or presence of 500 ng/ml rhPGRN for indicated time. The cell lysis was subjected to western blot for NFE2L2, p-NFE2L2 and GAPDH. **B** HeLa cells were transfected with PGRN siRNA for 48 h, NFE2L2 and p-NFE2L2 level was detected by western blotting. **C** HeLa cells were treated with or without PGRN and the interaction between KEAP1 and NFE2L2 was measured by co-IP assay. Ab: antibody. **D** HeLa cells were treated with serum-free medium for 12 h and then treated with 500 ng/mL rhPGRN in the presence or absence of rapamycin, LY294002 and U0126. The cell lysis was subjected to western blotting for NFE2L2, p-NFE2L2 and GAPDH. **E**, **F** HeLa cells were treated with or without rhPGRN and the localization of NFE2L2 was measured by western blotting (E) and IF (F) assay. **G** Cells were treated as described in (**E**), and the transcription activity of NFE2L2 was measured by ChIP assay. Ab: antibody. **H** Cells were treated as described in (**E**), and the NFE2L2 promoter activity was identified by luciferase reporter assay. **I**, **J** Cells were treated as described in (**E**), and the mRNA level of HO-1 (**I**) and GSTM (**J**) was measured by qRT-PCR. Data are presented as means ± SDs and are representative of 3 independent experiments. **P < 0.01; ***P < 0.001; ****P < 0.0001.

NFE2L2 is arguably the most important regulator of the expression of molecules with antioxidant roles within the cell through the antioxidant response element (ARE) [22]. NFE2L2 phosphorylation at Ser40 is necessary for the dissociation of NFE2L2 from KEAP1, a substrate adapter for a Cullin 3 (CUL3)-based E3 ubiquitin ligase that targets NFE2L2 for ubiquitination and proteasomal degradation, and nuclear translocation [23]. We then examined the effect of PGRN on NFE2L2 activity and found that rhPGRN treatment effectively stimulated NFE2L2 phosphorylation at Ser40 in HeLa cells subjected to serum deprivation (Fig. 6A). Meanwhile, knocking down the expression of PGRN resulted in decreased phosphorylation of NFE2L2 (Fig. 6B). Co-immunoprecipitation (co-IP) assay revealed that rhPGRN treatment markedly impaired the interaction between KEAP1 and NFE2L2 in HeLa cells with serum deprivation (Fig. 6C). We further determined the dependence of NFE2L2 phosphorylation on PGRN-stimulated signaling. As shown in Fig. 6D, inhibition of mTOR, PI3K/AKT and MEK/ERK signaling reduced rhPGRN-stimulated NFE2L2 phosphorylation in serum-deprived HeLa cells. As a transcription factor, NFE2L2 performs its functions in nuclear after dissociation from KEAP1. We found that rhPGRN-treatment led to an enhanced abundance of NFE2L2 in nuclear fraction of HeLa cells with serum deprivation (Fig. 6E). Immunofluorescence (IF) staining revealed that NFE2L2 was translocated from the cytoplasm to the nucleus in serum-deprived HeLa cells after rhPGRN treatment (Fig. 6F). The DNA-binding activity of NFE2L2 was then assessed in a chromatin immunoprecipitation (ChIP) assay. As shown in Fig. 6G, NFE2L2 antibody precipitated portions of the promoter of NQO-1 containing the ARE sequence in serum-deprived HeLa cells, and rhPGRN treatment enhanced the amount of NFE2L2-binding DNA. We next detected the effect of PGRN on the transcriptional activity of NFE2L2 under serum deprivation conditions by using a luciferase reporter system. A pGL6 construct containing ARE binding sites (p-ARE-luc) was transiently transfected into HeLa cells, and serum deprivation decreased the luciferase activity, which was rescued dose-dependently by rhPGRN treatment (Fig. 6H). Furthermore, the mRNA levels of NFE2L2-controlled HO-1 and GSTM were higher in serum-deprived HeLa cells with rhPGRN treatment than that of cells treated with serum deprivation alone (Fig. 6I, J).

### NFE2L2/ARE pathway mediates the protective role of PGRN in cervical cancer cells under serum deprivation conditions

**Fig. 7:**
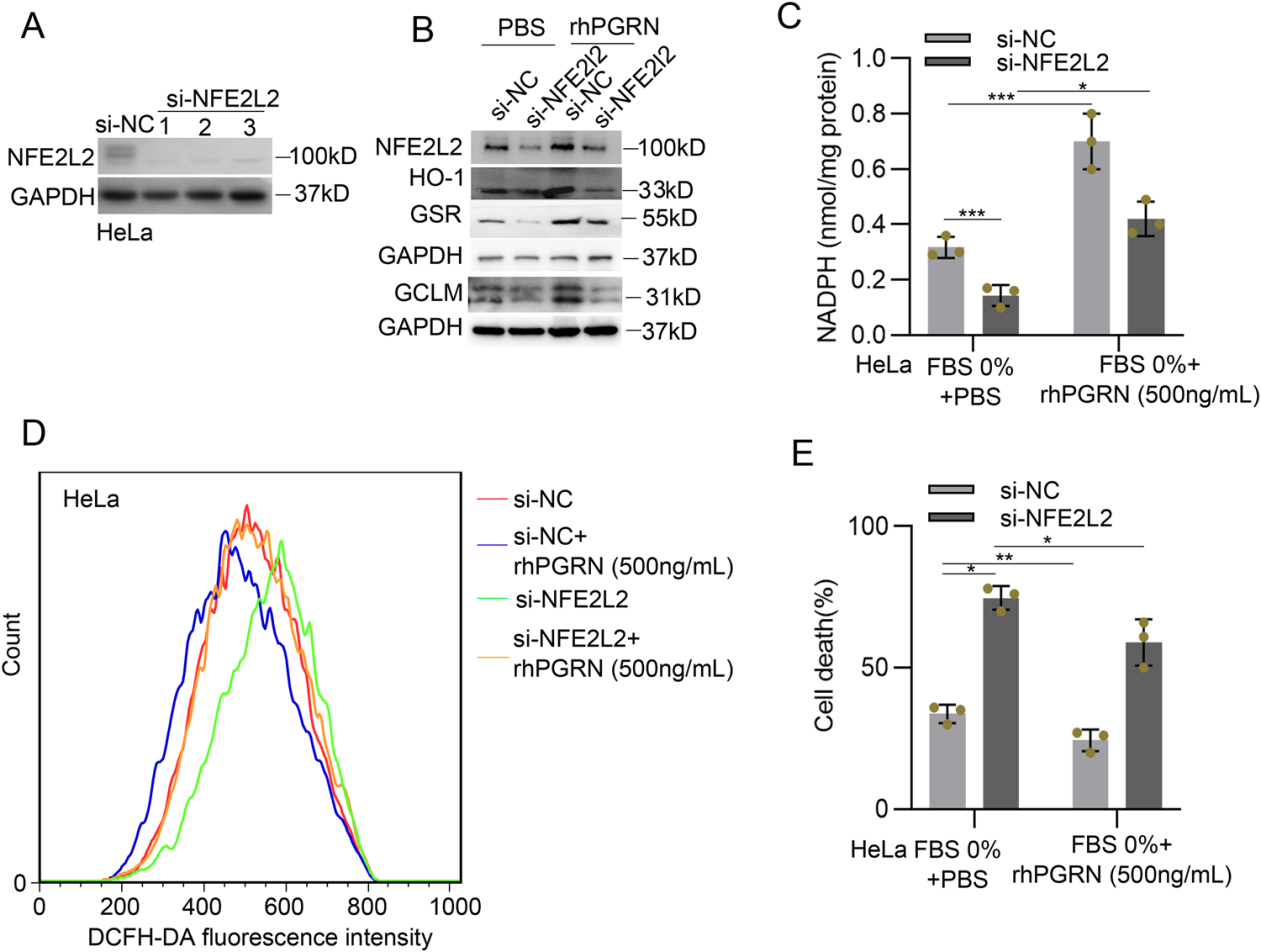
NFE2L2/ARE pathway mediated the protective role of PGRN in cancer cells under oxidative stress. **A** The efficiency of NFE2L2 knockdown was determined via western blotting. **B** HeLa cells were transfected with NFE2L2 siRNA for 48 h and then treated with serum deprivation medium in the absence or presence of 500 ng/mL rhPGRN and the indicated protein levels were measured by western blotting. **C** Cells were treated as in (**B**), and total NADPH level was measured. **D** Cells treated as in (**B**), and ROS level was determined by DCFH-DA. **E** Cells treated as in (**B**), cell death was determined by trypan blue assay. Data are presented as means ± SDs and are representative of 3 independent experiments. *P < 0.05; **P < 0.01; ***P < 0.001.

We further determined whether the antioxidant activity driven by PGRN is dependent on the NFE2L2/ARE pathway. Suppression of NFE2L2/ARE pathway by siRNA-mediated silencing of the NFE2L2 gene (Fig. 7A) effectively diminished PGRN-induced expression of antioxidant enzymes (Fig. 7B), and subsequently impaired PGRN-mediated enhancement of intracellular NADPH levels of serum-deprived HeLa cells (Fig. 7C). Under serum deprivation conditions, the inhibition of NFE2L2 expression impeded rhPGRN-mediated clearance of cellular ROS in HeLa cells (Fig. 7D). As a result, the protective role of PGRN in serum deprivation-induced cell death was disrupted by suppression of NFE2L2/ARE pathway in HeLa cells (Fig. 7E).

## Discussion

As a natural consequence of sustained proliferation, tumor cells are inevitably stripped of vascular supply, resulting in deprivation of oxygen and nutrients, which limits tumor growth. However, whether PGRN as a growth factor is involved in the survival of cervical cancer cells under these conditions remains to be further studied. In the current study, we revealed that compared to tissues adjacent to blood vessels, PGRN protein levels were decreased at sites far from blood vessels. This unique change drives us to investigate the reasons for the decrease in PGRN levels caused by insufficient blood supply and its role in the progression of cervical cancer.

Serum is a common supplement used in cell, tissue and organ cultures. It contains essential components such as hormones, vitamins and growth factors [24]. Given that cancer cells often suffer from insufficient support from local blood vessels, we used serum deprivation to mimic inadequate blood supply. In our study, we found that intracellular PGRN protein levels increased with increasing serum concentrations and decreased with decreasing serum concentrations. It has been reported that PGRN levels increase in rapidly proliferating epithelial cells [25]. Since serum provides an important stimulus signal for cell proliferation, this may explain why PGRN levels increase in the presence of serum and decreased in areas far from blood vessels in cervical cancer. Guerra et al reported that PGRN expression increases in response to hypoxia and low pH [18]. However, in our research, we found that only serum deprivation but not hypoxia and glucose deprivation can decrease the intracellular protein level of PGRN in cervical cancer cells, suggesting that compared with other stress factors, serum deprivation has a specific effect on PGRN expression in tumor microenvironments. Further studies revealed that increased secretion of PGRN is responsible for the decrease of intracellular PGRN levels under serum deprivation conditions.

The deficiency of growth factors, which is mimicked by serum deprivation, leads to decreased activity of the PI3K/AKT signaling pathway and diminished glucose uptake capacity of cells. This indirectly causes glucose metabolism defects and induces oxidative stress [6, 26]. In our study, we also found that serum deprivation promoted intracellular and mitochondrial ROS production, damages cellular components such as DNA, proteins, and lipids. Numerous studies have reported that PGRN promotes cell survival under various microenvironment stress conditions, including PGRN significantly improve neurons cell survival and cerebral ischemia-reperfusion injury by inhibit oxidative stress [27, 28], and our previous study has shown that PGRN can attenuate hypoxia-induced inflammatory actions and apoptosis in proximal tubule epithelial cells in murine models of acute kidney injury [19]. In this work, we also revealed that PGRN ameliorates the oxidative stress and subsequent lipid oxidation, protein oxidation and mitochondrial damage induced by serum deprivation.

To maintain the balance between ROS production and antioxidative defence, cells possess various antioxidant systems, including the antioxidant cofactors GSH, SODs and NAPDH [9]. In our research, we demonstrated that PGRN inhibits serum deprivation-induced ROS levels by promoting the activity of antioxidant enzymes including NQO-1, GSTM, TXN1, SOD, PRDX1 and GCLM. It is worth noting that most antioxidant enzymes, such as GSH, NQO-1, thioredoxin-1, TXNRD1 and peroxiredoxin1 are regulated by the master regulatory transcription factor NFE2L2. NFE2L2 promotes the expression of antioxidant-related genes by binding to the ARE of its downstream genes. NFE2L2 is expressed in all cell types and its basal protein levels are typically low under unstressed conditions mainly due to KEAP1-mediated proteasomal degradation. Under normal conditions, NFE2L2 is bound to KEAP1 and is continuously degraded by proteasome. Under oxidative stress conditions, the KEAP1-NFE2L2 complex is disrupted, leading to the nuclear translocation of NFE2L2, resulting in the transactivation of target genes [29, 30]. Our results showed that PGRN promotes the dissociation of NFE2L2 from KEAP1, which subsequently inhibits the ubiquitination-mediated degradation of NFE2L2 under serum deprivation conditions. Phosphorylation of NFE2L2 at Ser40 is necessary for NFE2L2 dissociation from KEAP1 [23]. Protein kinases, such as ERK [31], JNK [31], PI3K-AKT [32], PKC [33], and PERK [34], have been reported to mediate phosphorylation of NFE2L2 and increase its stability and subsequent transcriptional activity. Our result also showed that inhibiting the PI3K/AKT, mTOR and MEK/ERK pathways reduces NFE2L2 phosphorylation induced by PGRN under serum deprivation conditions. Although our previous study confirmed that PGRN can promote the phosphorylation of PKC [20], whether the effect of PGRN on the phosphorylation level of NFE2L2-Ser40 is dependent on the activation of PKC needs to be further analyzed. All these results suggest that the antioxidative effect of PGRN may depend on the NFE2L2/ARE signaling pathway activity and inhibiting NFE2L2 expression can suppress the antioxidative activity of PGRN in cervical cancer.

In conclusion, our study revealed that PGRN is a serum response gene that contributes to cell survival by eliminating ROS under serum deprivation conditions. Furthermore, our findings demonstrate for the first time that PGRN-mediated antioxidant activity is dependent on the activation of NFE2L2/ARE signaling. Inhibiting PGRN expression can suppress NFE2L2-related antioxidant protein expression, resulting in drastic oxidative stress that can induce cell death. Our understanding of PGRN-stimulated NFE2L2/ARE signaling not only characterizes the antioxidant role of PGRN under oxidative stress conditions but also provides new therapeutic interventions for PGRN in cervical cancer.

## Materials and methods

### Cell culture and treatments

Experiments were performed using the established human cervical cancer cell lines HeLa and SiHa. All cells were purchased from the American Type Culture Collection (ATCC) and cultured following the ATCC instructions. All the cells were cultured in DMEM medium containing 10% FBS, and 1% penicillin-streptomycin. All the cell lines were authenticated by short tandem repeat analysis and were tested for mycoplasma contamination. For serum starvation assay, cells were incubated with 0% FBS medium for 12 h and then stimulated with different concentration of FBS for 6 h, PGRN protein level was detected by western blotting.

### siRNA transfection

HeLa cells were seeded into 6-well culture plates and cultured in medium without antibiotics. Cells were transfected with short interfering RNA (siRNA) via the Lipofectamine 3000 reagent following the manufacturer’s protocol. The siRNA target sequences for human PGRN was 5′-GTGAGCTGCCCAGATGGCT-3′, and those for human NFE2L2 were 5′-TCCCGTTTGTAGATGACAA-3′, 5’-GAGAAAGAATTGCCTGTAA-3′ and 5′-CAGTCTTCATTGCTACTAA-3′. All siRNAs were purchased from Biosun Inc (Jinan, China).

### Cell survival assays

For cell proliferation assay, HeLa cells were seeded into 96-well plates at a volume of 100 μL per well. After 24 h, cells were incubated with complete DMEM medium, serum free DMEM, serum free DMEM plus 100 ng/mL rhPGRN, or serum free DMEM plus 500 ng/mL rhPGRN. Cell viability was measured using CCK-8 assays.

### ELISA of PGRN secretion

HeLa and SiHa cells were seeded into 6-well plates. After 12 h, cells were incubated with complete DMEM medium or serum free DMEM for indicated time and then medium was collected. PGRN secreted into the medium was detected using an ELISA kit (R&D Systems, MN, USA).

### Western blot analysis and immunoprecipitation

Western blot assays were performed as described previously [20]. Antibodies used for western blot analysis are summarized in Supplementary Table 1.

For immunoprecipitation, 800 μg of cell lysate was incubated with 8 μg of KEAP1 antibody for 12 h at 4 °C and then incubated with protein A/G magnetic beads overnight at 4 °C with constant rotation. After elution, the bound proteins were detected via western blotting with NFE2L2 antibody.

A nuclear/cytosol fractionation assay was performed using a ProteoExtarct subcellular proteome extraction kit (Merck, 539790) according to the manufacturer’s instructions. Briefly, cytoplasmic and nuclear extracts were prepared using the indicated buffer, respectively. Tubulin and SP1 were used as loading controls for the cytoplasmic and nuclear fractions.

### Real-time qRT-PCR

Total RNA was extracted from the cells via TRIzol reagent according to the manufacturer’s protocol (Vazyme Biotech, R401-01). One microgram of RNA was reverse-transcribed with ReverTra Ace qPCR RT kit (Toyobo, PCR-311). Real-time qPCR was performed using the SYBR Green mix (Toyobo, QPK-201). The primer sequences for the target genes were as follows: PGRN (forward, 5′-GGACAGTACTGAAGACTCTG-3′; reverse, 5′-GGATAACAGCTTGTAATGTG-3′), HO-1 (forward, 5′-AAGACTGCGTTCCTGCTCAAC-3′; reverse, 5′-AAAGCCCTACAGCAACTGTCG-3′), and GSTM (forward, 5′-TCTGCCCTACTTGATGGG-3′; reverse, 5′-TGCACCCAGGGAAGTGTGTTGTAT-3′).

### DAPI assay

Cell apoptosis in HeLa cells was detected by DAPI assay following the manufacturer’s protocols.

### Immunofluorescence staining

HeLa cells were seeded onto chamber slides and treated with serum free medium for 12 h and then incubated with 100 ng/mL or 500 ng/mL rhPGRN for 12 h. Immunofluorescence cell staining was performed as described. Briefly, cells on slides were incubated with the primary antibody for NFE2L2, followed by 40 min incubation of the secondary antibody in dark at 37 °C and images in the focal plane of the cell bodies were captured with the Zeiss confocal microscope.

For measurement the state of mitochondrial, HeLa cells were stained with Mito-Tracker Green (Beyotime, C1048) following the manufacturer’s protocols and images in the focal plane of the cell bodies captured with the Zeiss LSM980 confocal from the Medical Science and Technology Innovation Center at Shandong First Medical University.

### Detection of DNA ladder

HeLa cells were seeded at 3 × 10^5^ cells in a 60 mm flask and cultured for 24 h. The medium was changed to serum-free DMEM or serum free DMEM containing rhPGRN (100 or 500 ng/mL) and continually cultured for 24 h. DNA was extracted with the DNA Ladder Extraction Kit (Beyotime, C0007) according to the manufacturer’s instructions. DNA was dissolved in TE buffer, electrophoresed in a 1% agarose gel containing ethidium bromide and photographed under UV light.

### Determination of cellular oxidant production

HeLa cells were seeded in 6-well plate and cultured for 12 h in serum free medium and then treated with indicated rhPGRN. Intracellular ROS were detected by DCFH-DA (Beyotime, S0033S) as described in the manufacturer’s protocol. ROS assay were generated and analyzed on a BD FACS Calibur Cytometer with the included software.

MDA levels in cells were measured using a MDA assay kit (Beyotime, S0131S) according to the manufacturer’s instructions, the MDA content was measured by optical absorption at 532 nm and MDA concentration was calculated in μmol per gram of protein.

Cellular GSH content were measured using the GSH and GSSG assay kit (Beyotime, S0053) following the manufacturer’s instructions.

Cellular NADPH content were determined using a Amplite Colorimetric NADP and NADPH assay kit (AAT Bioquest, 15260) following the manufacturer’s protocol.

Protein oxidation was measured using an OxyBlot Oxidation Detection Kit (Millipore, S7150) following the manufacturer’s protocol.

SOD activity was measured using the total SOD assay kit with WST-8 (Beyotime, S0101S). According to the protocol, cell lysis was mixed with WST-8 and related buffers in 96-well plates, and incubated at 37 °C for 30 min. Optical absorption was measured at 450 nm.

The determination activity of catalase was performed according to the manufacturer’s instructions (Beyotime, S0051). Briefly, cells were lysed in lysis buffer and incubated on ice for 1 h. Cell lysis samples were mixed with the corresponding test solution. After 30 min, catalase activity was detected with a microplate analyzer at an absorbance of 520 nm.

### Measurement of ATP levels

HeLa cells were seeded onto 6-well plates and treated with serum free medium for 12 h and then incubated with 100 ng/mL or 500 ng/mL rhPGRN for 12 h. ATP levels were measured according to the manufacture’s instruction (Beyotime, S0026).

### Statistics

Data are expressed as the means ± SDs. The significance of the differences in mean values between and within multiple groups was examined by one-way analysis of variance followed by Duncan’s multiple range tests. *P* < 0.05 was considered statistically significant.

## Supplementary information

Supplementary information

Supplementary Table 1

RAW DATA

## Data Availability

The data supporting the findings of this study are available from the corresponding author upon reasonable request.
