# Supplementary material for: PGRN protects against serum deprivation-induced cell death by promoting the ROS scavenger system in cervical cancer
Source: Cell Death Dis. 2024 Dec 18;15(12):889. doi: 10.1038/s41419-024-07233-0 (PMC11655951; doi:10.1038/s41419-024-07233-0)
Supplement: Supplementary file 1 — Supplementary information [file 41419_2024_7233_MOESM1_ESM.doc]

**Supplementary information**

**
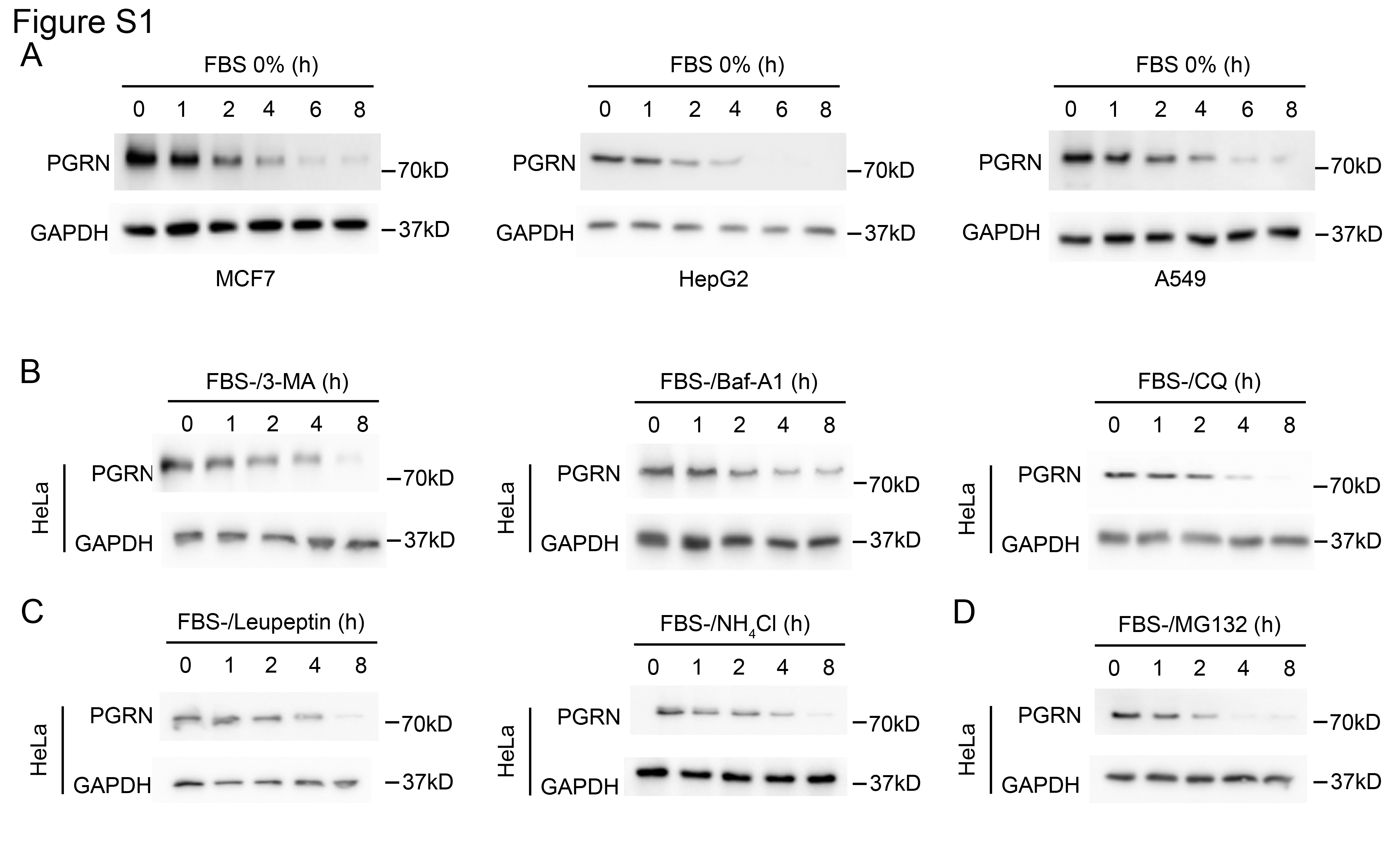
**

**Figure S1. Serum deprivation decreases PGRN protein levels independent of autophagy, lysosomal and lysosomal protease degradation.**

**A,** MCF-7, HepG-2 and A549 cells were treated with serum deprivation for indicated time and intracellular PGRN protein levels was analyzed by western blotting. **B,** HeLa cells were treated with serum deprivation in the presence of autophagy inhibitors 3-MA, Baf-A1 or CQ for the indicated time. Intracellular PGRN protein levels were analyzed by western blotting. **C,** HeLa cells were treated with serum deprivation in the presence of lysosomal protease inhibitor leupeptin and lysosomal inhibitor NH_4_Cl for indicated time. Intracellular PGRN protein levels were analyzed by western blotting. **D,** HeLa cells were treated with serum deprivation in the presence of proteasome inhibitors MG132 for indicated time. Intracellular PGRN protein levels were analyzed by western blotting.

**
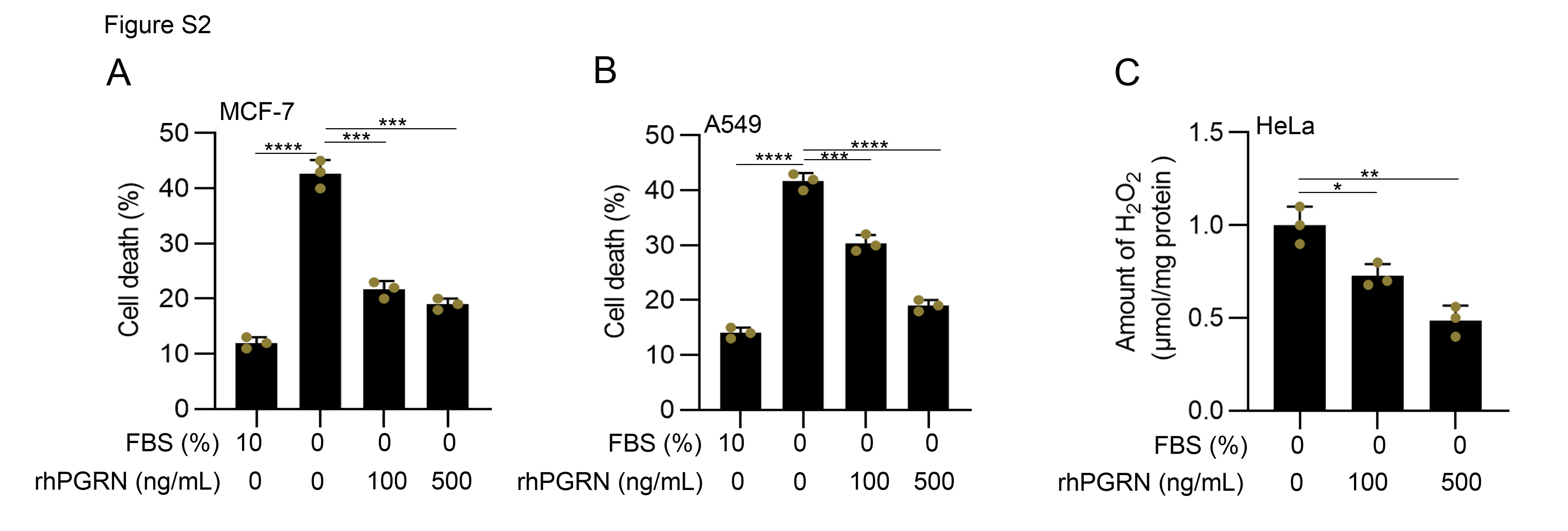
**

**Figure S2. PGRN inhibits cancer cell death under serum deprivation conditions.**

**A-B,** MCF-7 (A) and A549 (B) cells were treated with serum deprivation for 12 h and then treated with rhPGRN. Cell death was determined by trypan blue staining. **C,** HeLa cells were treated with serum deprivation for 12 h and then treated with indicated concentration of rhPGRN. The H_2_O_2_ levels were determined by H_2_O_2_ assay. Data are presented as means ± SDs and are representative of 3 independent experiments. **P*<0.05; ***P*<0.01;****P*<0.001; *****P*＜0.0001.

**
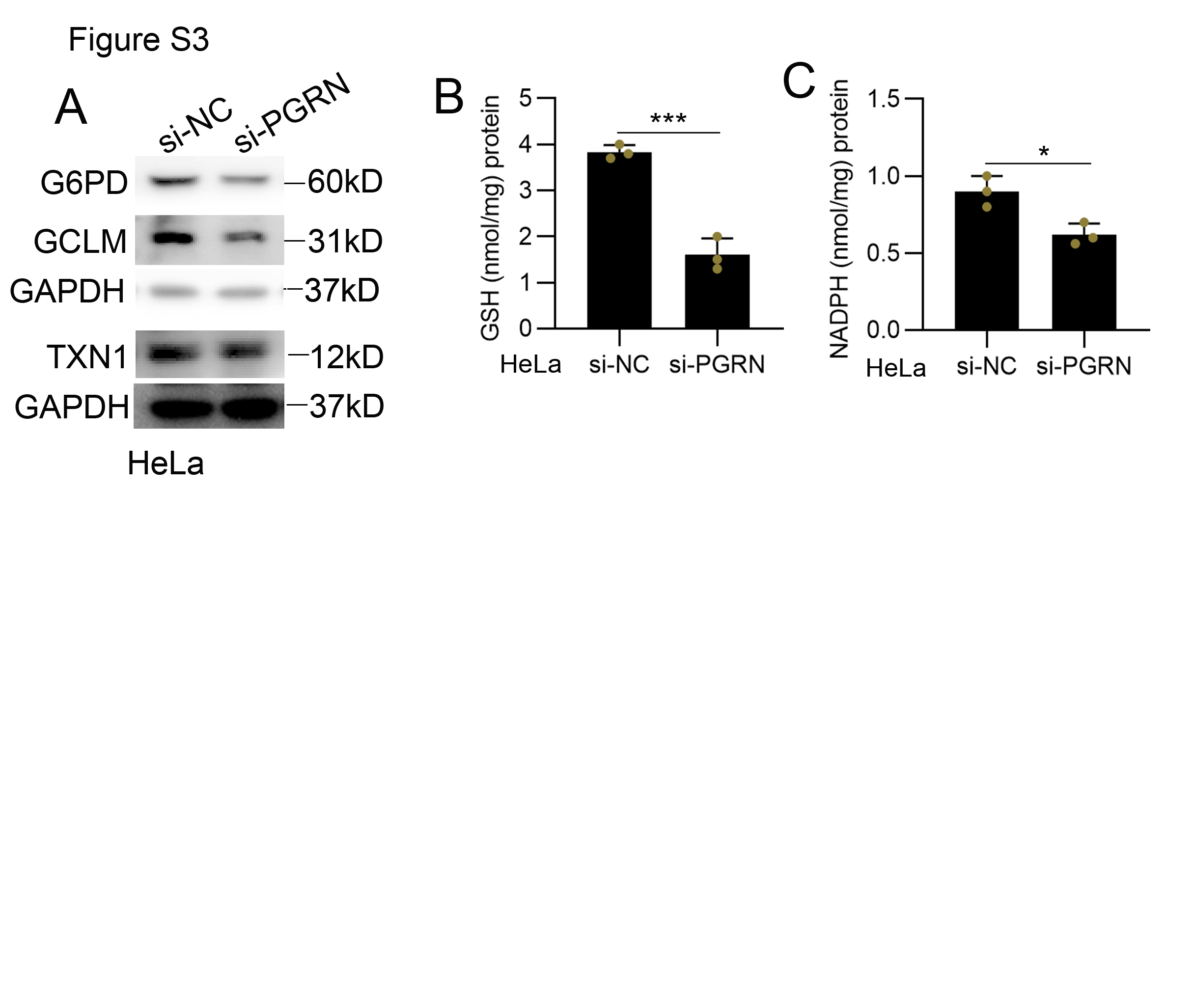
**

**Figure S3. Inhibition of PGRN expression decreases the level of the ROS scavenger.**

**A,** HeLa cells were transfected with siPGRN for 48 h, indicated protein level were analyzed by western blotting. **B-C,** HeLa cells treated as in (A), and then GSH (B) and total NADPH (C) level was measured. Data are presented as means ± SDs and are representative of 3 independent experiments. **P*<0.05, ****P*<0.001.
