# Supplementary material for: PGRN protects against serum deprivation-induced cell death by promoting the ROS scavenger system in cervical cancer
Source: Cell Death Dis. 2024 Dec 18;15(12):889. doi: 10.1038/s41419-024-07233-0 (PMC11655951; doi:10.1038/s41419-024-07233-0)
Supplement: Supplementary file 2 — Supplementary Table 1 [file 41419_2024_7233_MOESM2_ESM.docx]

| Primary antibodies | Host | Dilution and supplier | Application |
| --- | --- | --- | --- |
| PGRN | Rabbit | 1:800; Santa Cruz | IHC |
| PGRN | Rabbit | 1:1000; Affinity | WB |
| PARP1 | Rabbit | 1:1000; Protein Tech Group | WB |
| BAX | Rabbit | 1:1000; Protein Tech Group | WB |
| HO-1 | Rabbit | 1:1000; Protein Tech Group | WB |
| GSTM | Rabbit | 1:1000; Protein Tech Group | WB |
| GCLM | Rabbit | 1:1000; Protein Tech Group | WB |
| GSR | Rabbit | 1:1000; Protein Tech Group | WB |
| TXN1 | Rabbit | 1:1000; Protein Tech Group | WB |
| PRDX1 | Rabbit | 1:1000; Protein Tech Group | WB |
| TXNRD1 | Rabbit | 1:1000; Protein Tech Group | WB |
| NFE2L2 | Rabbit | 1:1000; Protein Tech Group | WB, IF |
| NQO-1 | Rabbit | 1:1000; Protein Tech Group | WB |
| G6PD | Rabbit | 1:1000; Protein Tech Group | WB |
| PGD | Rabbit | 1:1000; Protein Tech Group | WB |
| ME1 | Rabbit | 1:1000; Protein Tech Group | WB |
| IDH1 | Rabbit | 1:1000; Protein Tech Group | WB |
| KEAP1 | Rabbit | 1:1000; Protein Tech Group | WB |
| Tubulin | Rabbit | 1:1000; Protein Tech Group | WB |
| p-ATM(Ser1981) | Rabbit | 1:800; Cell Signaling technology | WB |
| p-Chk1(Ser345) | Rabbit | 1:800; Cell Signaling technology | WB |
| SP1 | Rabbit | 1:1000; Cell Signaling technology | WB |
| p-NFE2L2(Ser40) | Rabbit | 1:800; MCE | WB |
| T-ATM | Rabbit | 1:800; Protein Tech Group | WB |
| T-Chk1 | Rabbit | 1:800; Protein Tech Group | WB |
| GAPDH | Rabbit | 1:3000; Protein Tech Group | WB |

Supplementary Table 1. Antibodies used in this study
