# Supplementary material for: PGRN protects against serum deprivation-induced cell death by promoting the ROS scavenger system in cervical cancer
Source: Cell Death Dis. 2024 Dec 18;15(12):889. doi: 10.1038/s41419-024-07233-0 (PMC11655951; doi:10.1038/s41419-024-07233-0)

Figure 2

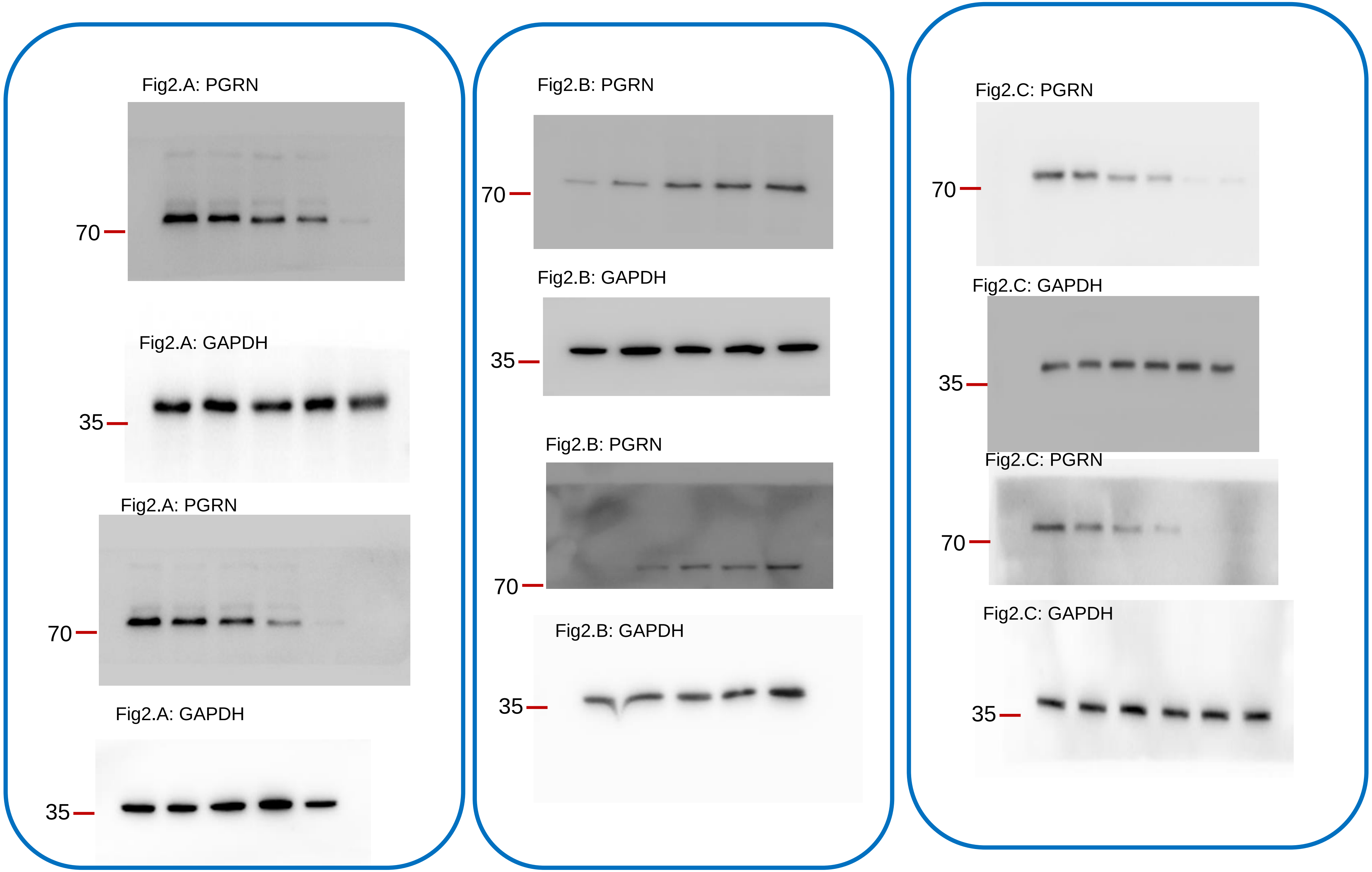

Fig2.A: PGRN
70
Fig2.A: GAPDH
35
Fig2.A: PGRN
70
Fig2.A: GAPDH
35
Fig2.B: PGRN
70
Fig2.B: GAPDH
35
Fig2.B: PGRN
70
Fig2.B: GAPDH
35
Fig2.C: PGRN
70
Fig2.C: GAPDH
35
Fig2.C: PGRN
70
Fig2.C: GAPDH
35

Figure 2

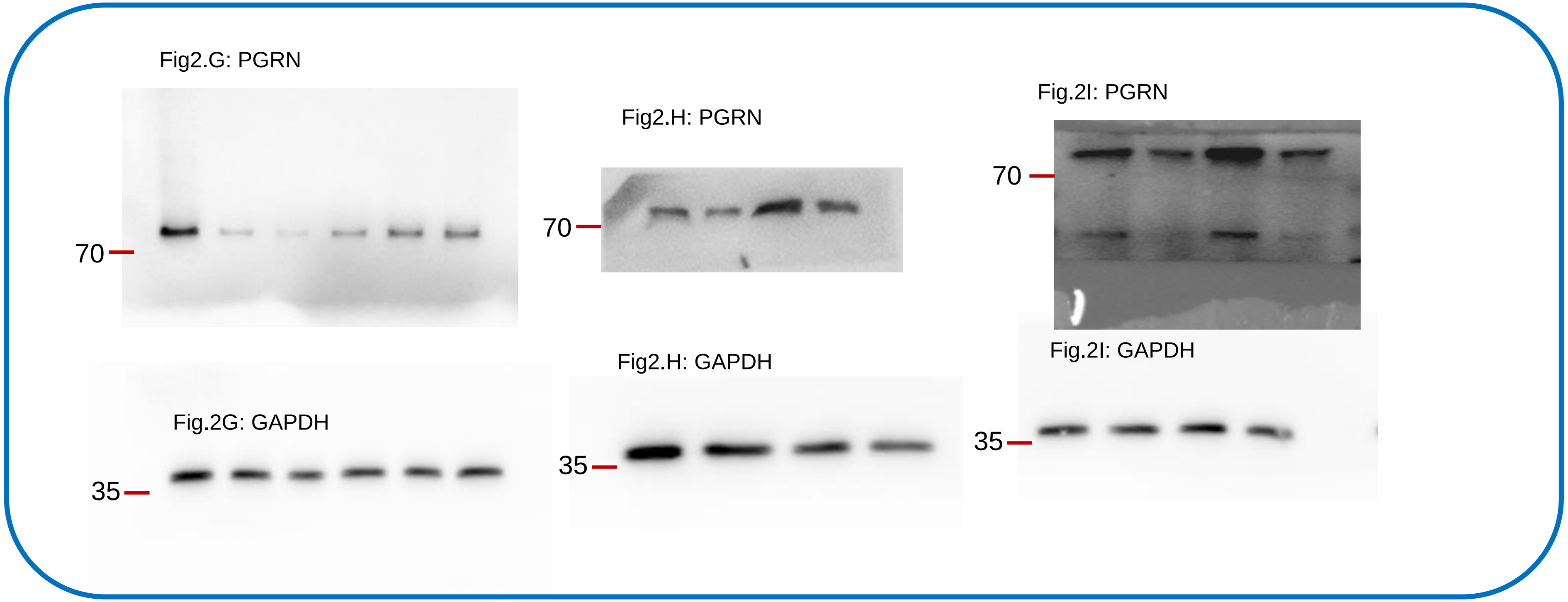

Fig2.G: PGRN
70
Fig2.H: PGRN
70
Fig.2I: PGRN
70
Fig.2G: GAPDH
35
Fig2.H: GAPDH
35
Fig.2I: GAPDH
35

Figure 3

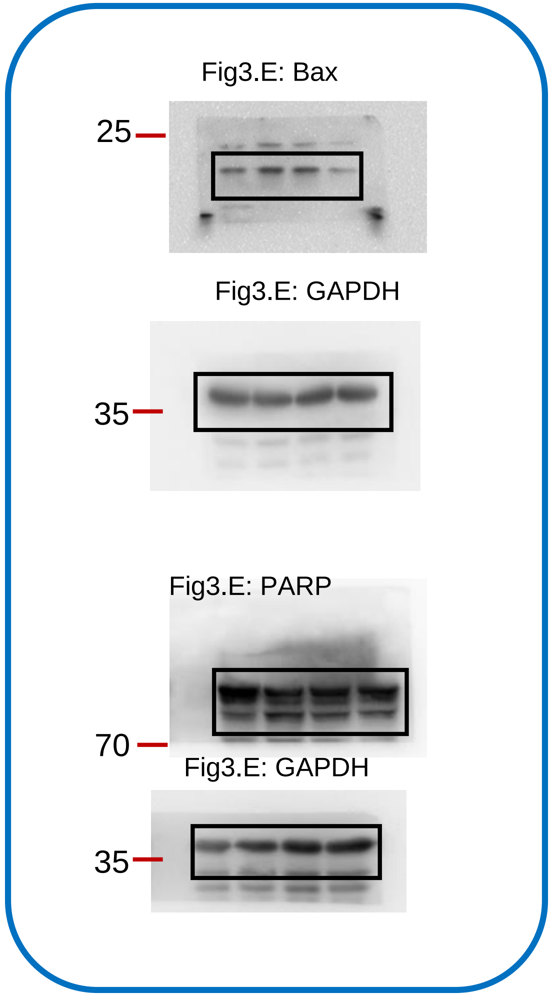

Fig3.E: Bax
25
Fig3.E: GAPDH
35
Fig3.E: PARP
70
Fig3.E: GAPDH
35

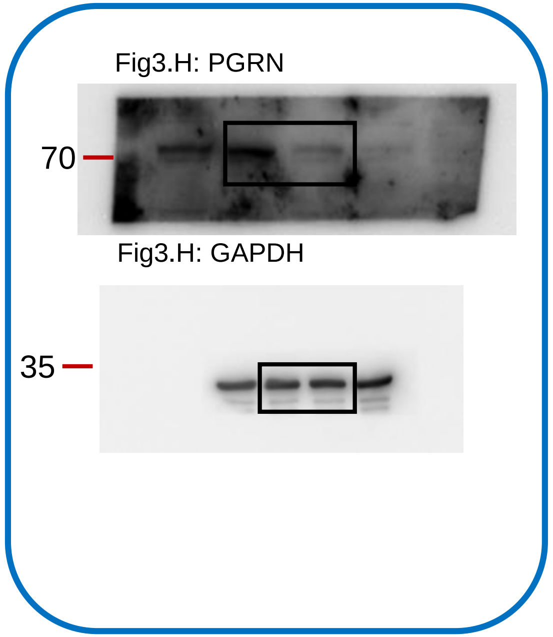

Fig3.H: PGRN
70
Fig3.H: GAPDH
35

Figure 4

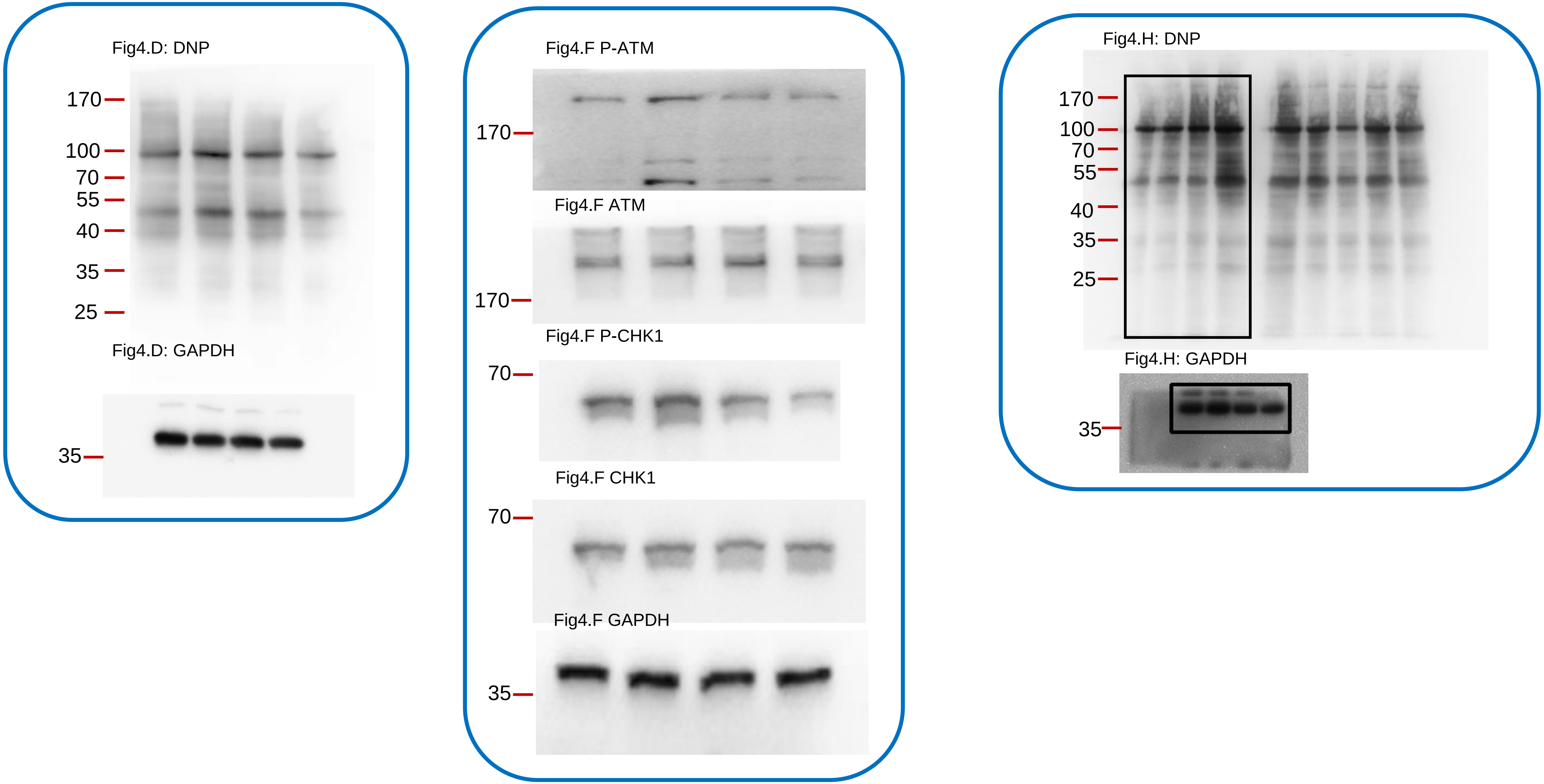

Fig4.D: DNP
170
100
70
55
40
35
25
Fig4.D: GAPDH
35
Fig4.F P-ATM
170
Fig4.F ATM
170
Fig4.F P-CHK1
70
Fig4.F CHK1
70
Fig4.F GAPDH
35
Fig4.H: DNP
170
100
70
55
40
35
25
Fig4.H: GAPDH
35

Figure 5

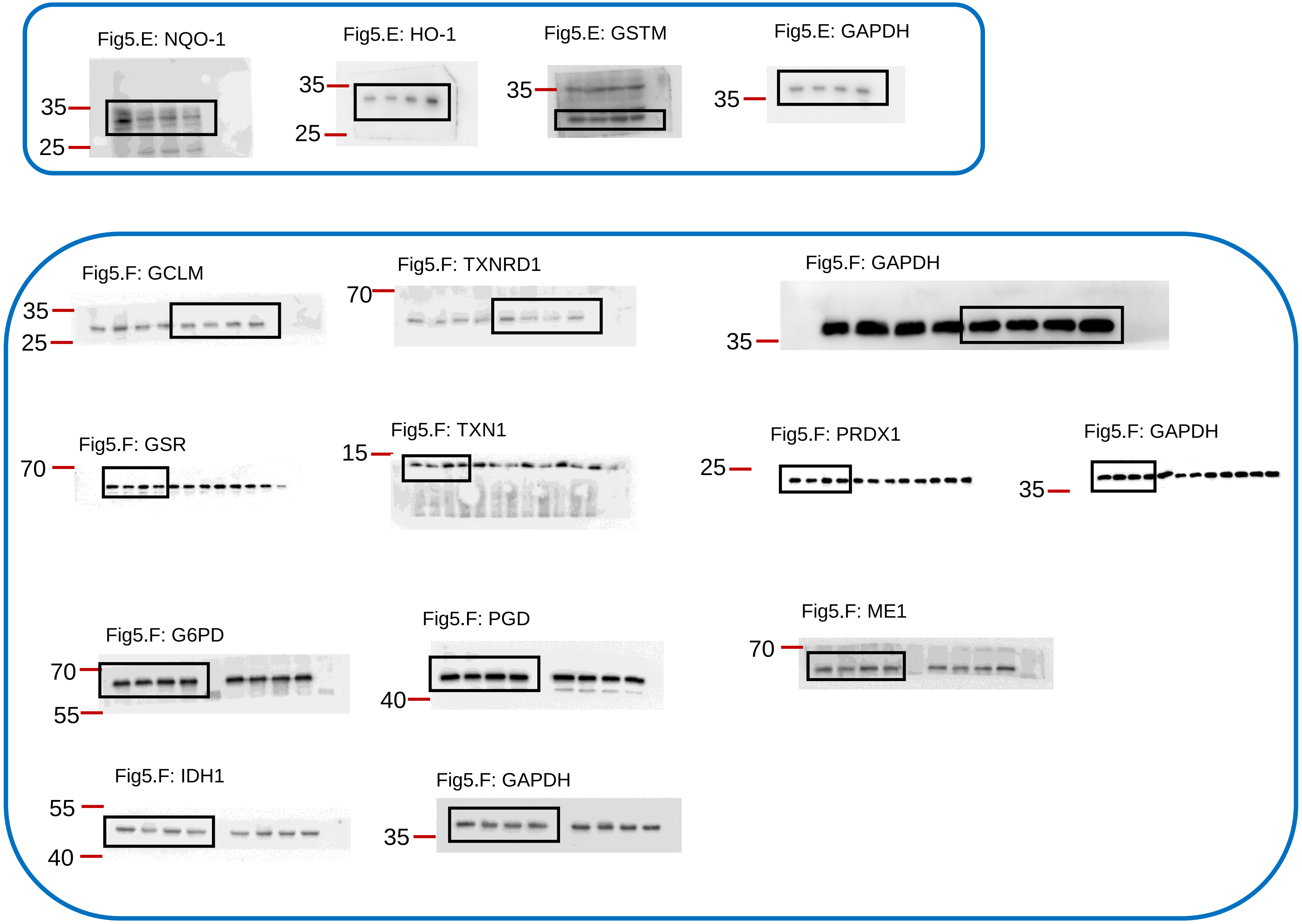

Fig5.E: NQO-1
35
25
Fig5.E: HO-1
35
25
Fig5.E: GSTM
35
Fig5.E: GAPDH
35
Fig5.F: GCLM
35
25
Fig5.F: TXNRD1
70
Fig5.F: GAPDH
35
Fig5.F: GSR
70
Fig5.F: TXN1
15
Fig5.F: PRDX1
25
Fig5.F: GAPDH
35
Fig5.F: G6PD
70
55
Fig5.F: PGD
40
Fig5.F: ME1
70
Fig5.F: IDH1
55
40
Fig5.F: GAPDH
35

Figure 6

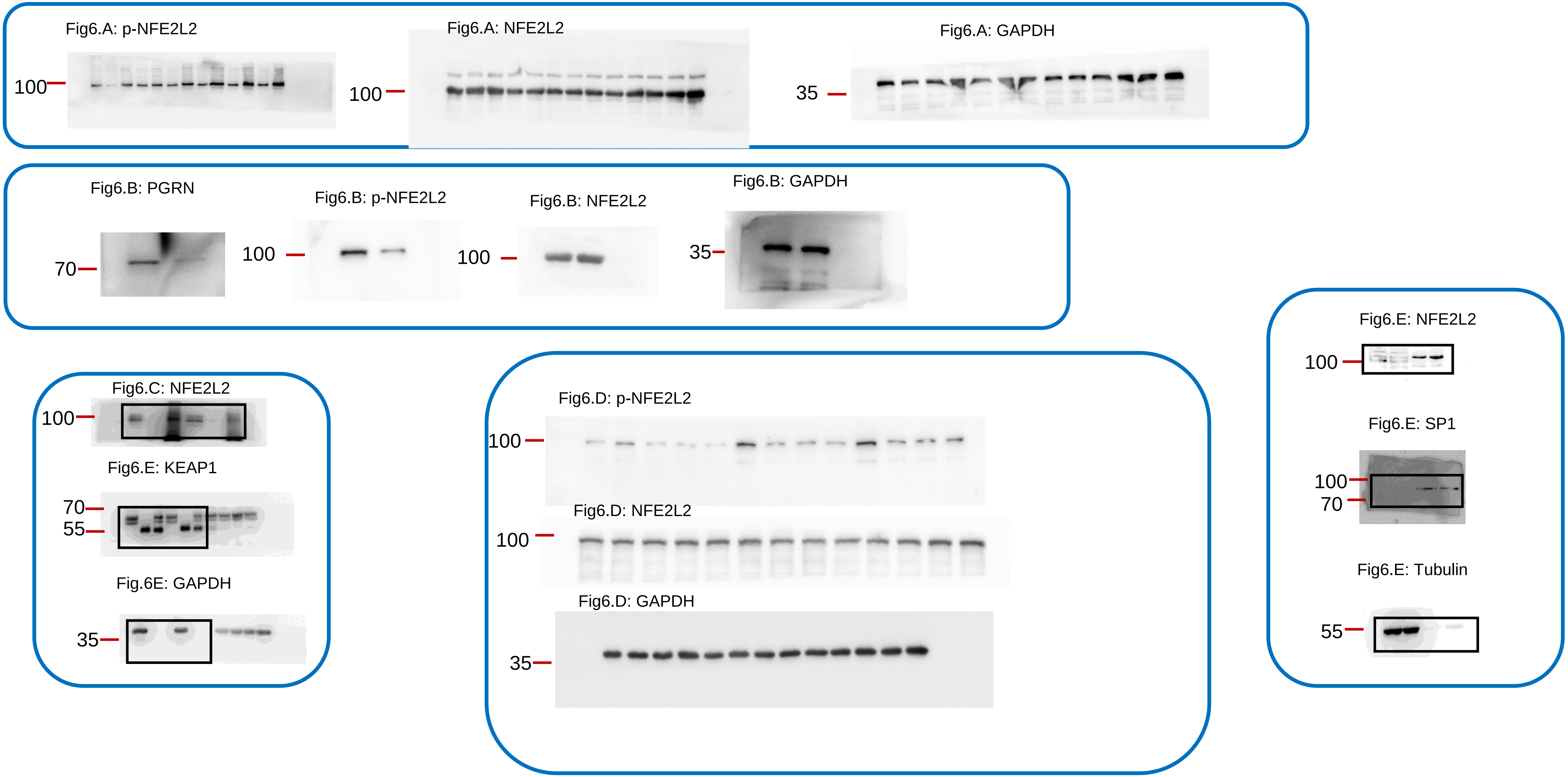
Fig6.A: p-NFE2L2
100
Fig6.A: NFE2L2
100
Fig6.A: GAPDH
35
Fig6.B: PGRN
70
Fig6.B: p-NFE2L2
100
Fig6.B: NFE2L2
100
Fig6.B: GAPDH
35
Fig6.C: NFE2L2
100
Fig6.E: KEAP1
70
55
Fig.6E: GAPDH
35
Fig6.D: p-NFE2L2
100
Fig6.D: NFE2L2
100
Fig6.D: GAPDH
35
Fig6.E: NFE2L2
100
Fig6.E: SP1
100
70
Fig6.E: Tubulin
55

Figure 7

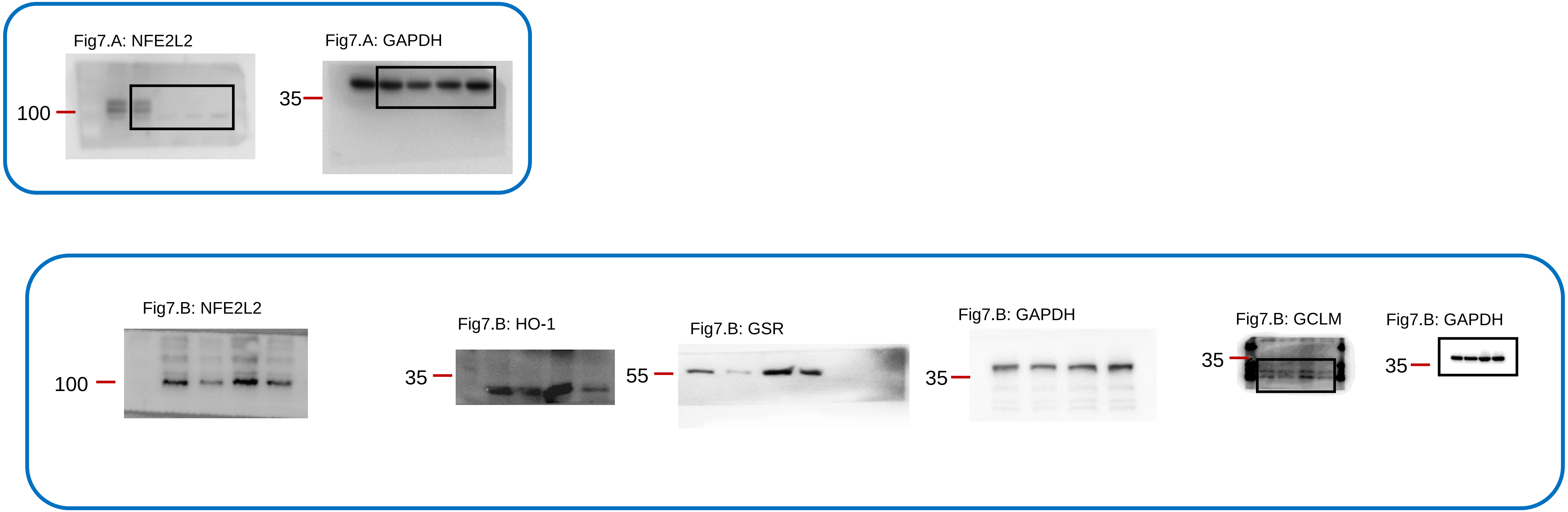

Fig7.A: NFE2L2
100
Fig7.A: GAPDH
35
Fig7.B: NFE2L2
100
Fig7.B: HO-1
35
Fig7.B: GSR
55
Fig7.B: GAPDH
35
Fig7.B: GCLM
35
Fig7.B: GAPDH
35

Supplementary figure 1

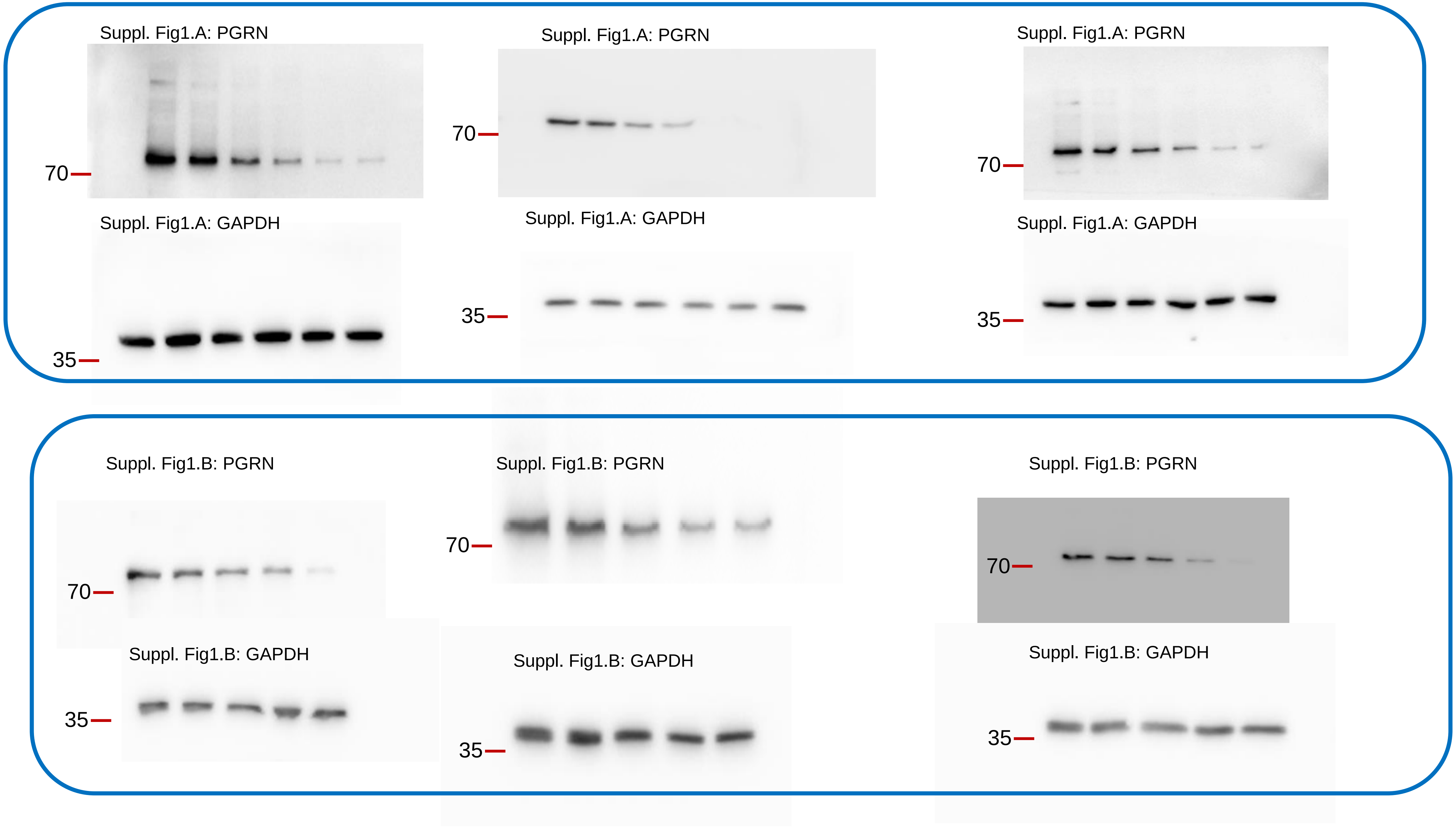

Supplementary figure 1

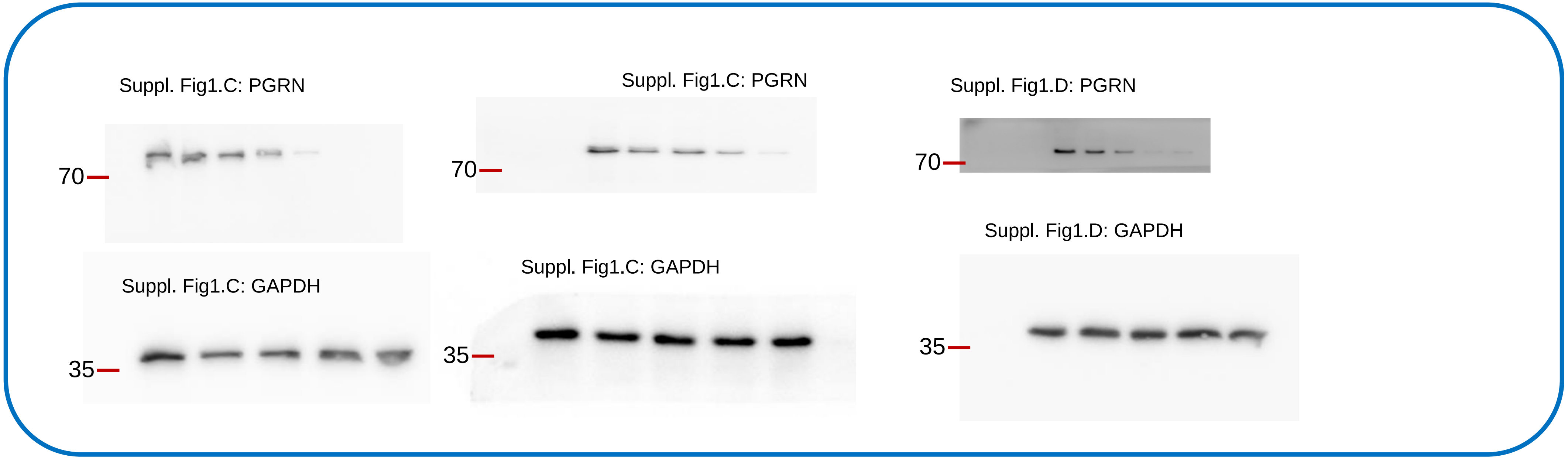

Supplementary figure 3

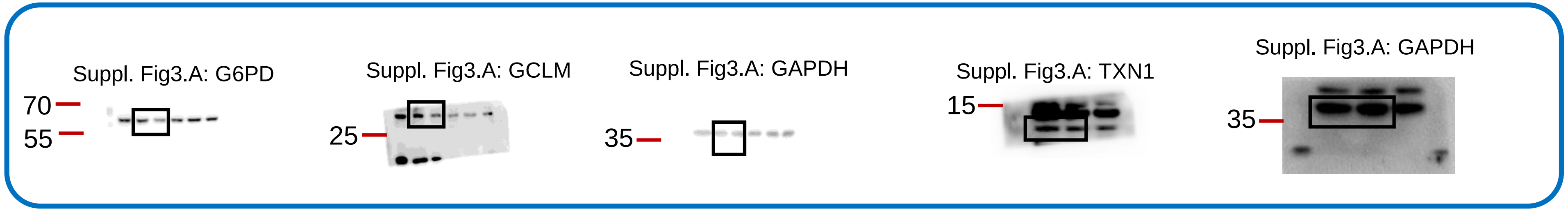

Supplement: Supplementary file 3 — RAW DATA [file 41419_2024_7233_MOESM3_ESM.pdf]
